# Biomimetic Pyramid Structure Film for Enhancing Building Radiative Cooling

**DOI:** 10.1002/advs.202413559

**Published:** 2025-02-14

**Authors:** Qian‐Hao Pan, Mei‐Hua Wang, Zong‐Ying Huang, Xiao‐Jing Qiu, Yu‐Tao Wang, Fu‐Xing Zhao, Meng‐Han Zhu, Xin Guo, Chen Chen, Si‐Chao Zhang, Jin‐Long Wang, Zhen He, Shu‐Hong Yu

**Affiliations:** ^1^ Shenzhen Key Laboratory of Sustainable Biomimetic Materials Department of Materials Science and Engineering Guangdong Provincial Key Laboratory of Sustainable Biomimetic Materials and Green Energy Institute of Innovative Materials Guangming Advanced Research Institute Southern University of Science and Technology Shenzhen 518055 China; ^2^ Department of Chemistry University of Science and Technology of China Hefei 230026 China; ^3^ New Cornerstone Science Laboratory Division of Nanomaterials & Chemistry Hefei National Research Center for Physical Sciences at the Microscale Department of Chemistry Institute of Biomimetic Materials & Chemistry Anhui Engineering Laboratory of Biomimetic Materials University of Science and Technology of China Hefei 230026 China

**Keywords:** biomimetic, building thermal management, environmental stability, pyramid structure, radiative cooling

## Abstract

Radiative cooling has emerged as a promising technique for reducing energy consumption in building thermal management due to its passive cooling property and no external energy requirement. Despite significant advances, scalable production of artificial photonic radiators with periodic structures, environmental stability, high radiative cooling performance, and economic applicability is still challenging in most state‐of‐the‐art radiative coolers. Rational structure and materials design are essential to promote daytime sunlight reflectance while maintaining a high emissivity within the atmospheric window (8–13 µm). In this work, inspired from the unique hair structure of heat‐resistant organisms, a biomimetic micro‐pyramid shaped structure model is analyzed. By mimicking the intricate design with a silicon template, a radiative cooling film containing specialized micro‐pyramid structure is fabricated by integrating high dielectric constant materials with polymers and receiving PVDF coating. The resulting film boasts a solar reflectance of 97.3% and an exceeding 98% infrared light emission within the atmospheric window. In addition, silicon rubber endows this membrane with strong tensile and rebound properties while surficial hydrophobicity protects the membrane from dust infestation. Considering the manufacturing simplicity and cost‐effectiveness, this method shows great potential for mass production, shedding light on building thermal management.

## Graphical abstract

Taking inspirations from the special surface structure of natural heat‐resistant organisms, a biomimetic micro‐pyramid‐shaped radiative cooling film is fabricated by integrating high dielectric constant materials with polymers. The resulting film boasts a solar reflectance of 97.3%, an exceeding 98% infrared light emission within the atmospheric window and achieves an average temperature drop of 7.3 °C for the coated device under sunlight irradiation.

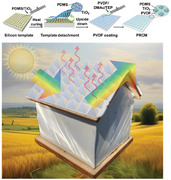

## 1 Introduction

Global warming is accelerating at an alarming rate and becoming irreversible at current stage, posing a substantial threat to the lives on earth.^[^ ^1^, ^2^, ^3^ ^]^ Over the years, approximately 20% of global carbon emissions is reported annually due to building cooling.^[^ ^4^ ^]^ However, traditional energy‐saving methods, such as photovoltaic, hydroelectric, and wind‐driven electric cooling, are limited in further applications due to the inferior conversion effect, enormous space consumption, time, region, and climate restrictiveness.^[^ ^5^ ^]^ It is essential to develop new cooling strategies with sustainable energy‐saving and low‐carbon thermal management models that are easy to integrate with a wide range of buildings.^[^ ^6^ ^]^ Radiative cooling, a rapidly emerging strategy in recent years, can effectively emit heat through the atmospheric infrared window to the cold universe and conduct heat exchange with outer space, resulting in temperature decrement inside buildings.^[^ ^7^, ^8^, ^9^ ^]^ Compared with these traditional energy‐saving methods, passive thermal radiation exhibits excellent features of zero‐energy consumption, passiveness, and sustainability, which show broader prospects in the future.^[^ ^10^, ^11^ ^]^ To sustain a steady daytime radiative cooling efficiency, the utmost reflection of solar energy and strong emissivity within 8–13 µm atmospheric windows need to be met simultaneously.^[^ ^12^, ^13^, ^14^ ^]^ Therefore, precise morphology design and size control are considered for manufacturing radiative cooling materials.^[^ ^15^, ^16^, ^17^, ^18^ ^]^ Pioneering works for high‐performance radiative coolers, including multilayer reflectors,^[^ ^19^ ^]^ photonic structure metamaterials, nanoparticle‐based radiators,^[^ ^20^ ^]^ plastic textiles,^[^ ^21^ ^]^ and hierarchical structure woods,^[^ ^22^ ^]^ have been developed recently. However, polyester and polyolefin‐based membranes tend to degrade and turn yellow when prolonged exposure to ultraviolet (UV) light, heat, water, and ambient chemicals (e.g., NO_x_, SO_x_, and O_3_).^[^ ^14^ ^]^ Solar absorption consequently increased, and materials could even change from cooling to heating in the daytime. Suffering from a complex preparation procedure and high costs, the multi‐layer designed membranes face challenges in mass production. Moreover, porous polymer coatings and foams are easily disturbed by environmental dust. Therefore, requirements need to be qualified simultaneously for a preferable radiative cooling film including: (1) High reflectivity for 0.3‐2.5 µm light and strong emissivity within 8–13 µm atmospheric windows, (2) Anti‐ultraviolet aging and dust disturbance, and (3) rapid mass production.

The diverse heat‐resistant organisms, including coleopteran insects,^[^ ^23^, ^24^, ^25^, ^26^, ^27^ ^]^ aquatic organisms,^[^ ^28^ ^]^ mammals,^[^ ^29^ ^]^ and arthropods found in extreme environments, have inspired the development of thermal management strategies.^[^ ^30^, ^31^, ^32^, ^33^, ^34^ ^]^ Thanks to the astonishingly ordered photonic structure on the surface,^[^ ^35^ ^]^ these organisms demonstrate incredible survivability in environments exceeding 50 °C.^[^ ^32^ ^]^ For instance, the distinctive surficial hair's structure of *C. Bombycina* is instrumental in maintaining proper temperature in hot environments. The hair's triangular cross‐section structure enhances the total reflection of light, while the pyramid‐like structure facilitates Mie scattering of light at the hair's interface, reducing the light intensity transmitted to the organism. These surface‐located, ordered periodic photonic structures significantly enhance solar reflection and thermal stability, providing a basis for novel solutions in radiative cooling technologies.^[^ ^36^, ^37^, ^38^ ^]^ According to the Bragg's diffraction theory, the periodic structure determines the lattice constant of the photonic crystal, which has a significant impact on light wavelength modulation, while the refractive index of the material determines the diffraction bandwidth and reflectivity of the light. The heat‐resistant biological model from nature provides structural references for excellent design of radiative cooling materials. However, higher requirements on material design have also been put forward. Owing to its excellent mid‐infrared emissivity, polymer materials such as polyethylene (PE), polyethylene terephthalate (PET), and polydimethylsiloxane (PDMS) have shown broad prospects as a supporting foundation for composite materials.^[^ ^39^ ^]^ In addition, using high dielectric constant particles has been theoretically and experimentally proved effective for radiative coolers. Thus, polymer‐dielectric composite film is supposed to demonstrate an advanced radiative cooling performance by doping the high dielectric constant particles into polymers.^[^ ^40^, ^41^ ^]^ Moreover, As an anti‐ultraviolet modification layer, PVDF is critical in enhancing ultraviolet reflection and anti‐aging, especially for 300–380 nm light. Coating a layer of PVDF on the composite film would further increase UV light reflection.

A biomimetic micro‐pyramid‐shaped radiative cooling film is fabricated in this work by analyzing the special hair structure of the heat‐resistance models. High solar light reflection is supposed to be accessible on the membrane as the pyramidal microstructure enhances the total reflection. In view of the flexibility of the membrane, it is supposed to be feasible for integration into building.

## 2 Results and Discussion

### 2.1 Morphology and Structure Characteristics of the Heat‐Resistant Organisms

**Figure 1:**
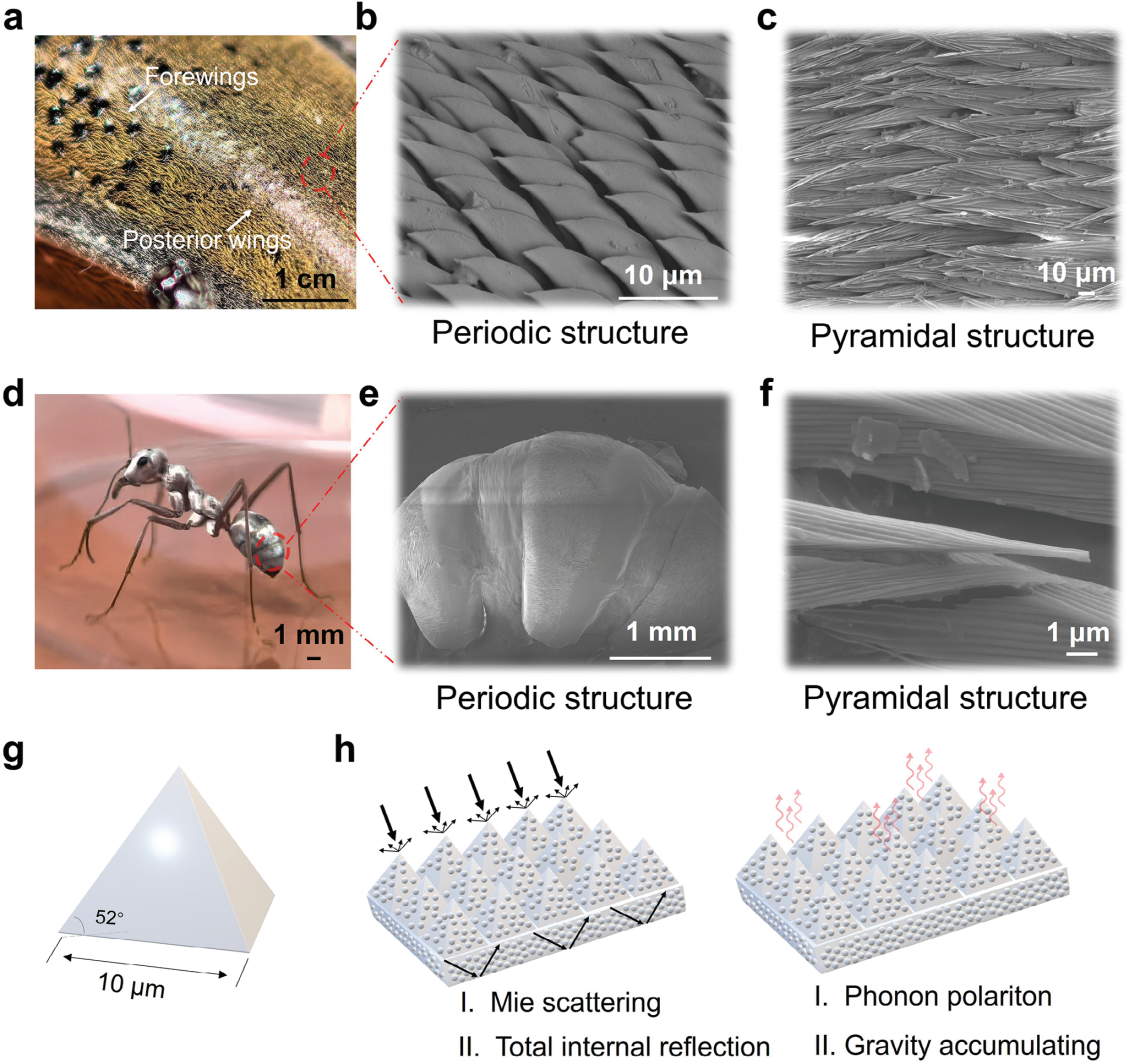
Morphology characterization of the *A. germari* and *C. bombycina*. a) Optical image of the *A. germari*. b, c) SEM morphology of *A. germari* posterior wings and forewings. Ordered periodically pyramidal structures are clearly to be observed. d) Optical image of the *C. bombycina*. e, f) SEM image of the *C. bombycina*. g) Schematic illustration of the biomimic micro‐pyramid. The ideal length of the micro‐pyramid bottom side is 10 µm and the elevation is 52°. h) Schematic demonstration of the micro‐pyramid structure for radiative cooling.

Microstructure characterizations were first applied to explore the structure‐induced mechanism of the heat‐resistant insects. Specimens of *A. germari* were acquired from field collection in Hainan province, China. The specimens were first soaked into 10% formalin solution over 6 hours for biological microstructure fixture. As illustrated in **Figure** **1**a, the forewings and posterior wings are tightly adhered to each other on the dorsal side of the longicorn beetle. The textures of the hairs grown on the *A. germari* allow light to be reflected strongly in specific directions, and a shiny golden surface emerges on this model. By carefully separating these wings and observing under scanning electron microscopy (SEM) the microscopic structures are discovered as illustrated in Figure 1b. The posterior wings are densely covered by periodic pyramidal structures with a height of around 5 µm and a length of 10 µm. Forewings feature hierarchical acicular fluffs that are oriented in the same direction. Fluffs are stacked on each other, and several well‐arranged wrinkles are observed on the surface (Figure 1c). The wrinkled surface area helps the *A. germari* reflect sunlight to survive hot summer days. A similar micro‐structure is also observed in the *C. bombycina*. As one of the most heat‐resistant organisms in the world, these silver ants maintain a stable “operative environmental temperature” to support their foraging activities even when the environmental temperature reaches over 60 °C. As the physical image shown in Figure 1d, the dorsal and lateral sides of the *C. bombycina* body demonstrate a silvery appearance. As the SEM images shown in Figure 1e,f, a periodical triangular structure is revealed locally aligned in the same direction. Cross‐sectional image obtained by the focused ion beam (FIB) milling (Figure S1, Supporting Information) further demonstrates the triangular profile of the hair. Due to this special structure of fluffs, silver ants exhibit a total internal reflection, which results in a higher UV–vis light reflection. Inspired from these two biological structure models, a simplified typical pyramidal structure model is proposed (Figure 1g) and the structure‐enhanced Mie scattering are supposed to gain high reflectivity in the vis‐NIR region (Figure 1h). Besides, inspired by the triangular section with densely distributed pleats on the bottom facet of the biological *C. bombycina*, the high emissivity in the MIR range can be achieved by gradually changing the refractive index. Considering that radiation cooling is mainly related to sunlight reflection and atmospheric window emission, the pyramid radiative cooling membrane (PRCM) with periodic structure is promising to develop new radiative cooling materials for building thermal management.

### 2.2 Materials and Structure Design of the PRCM

**Figure 2:**
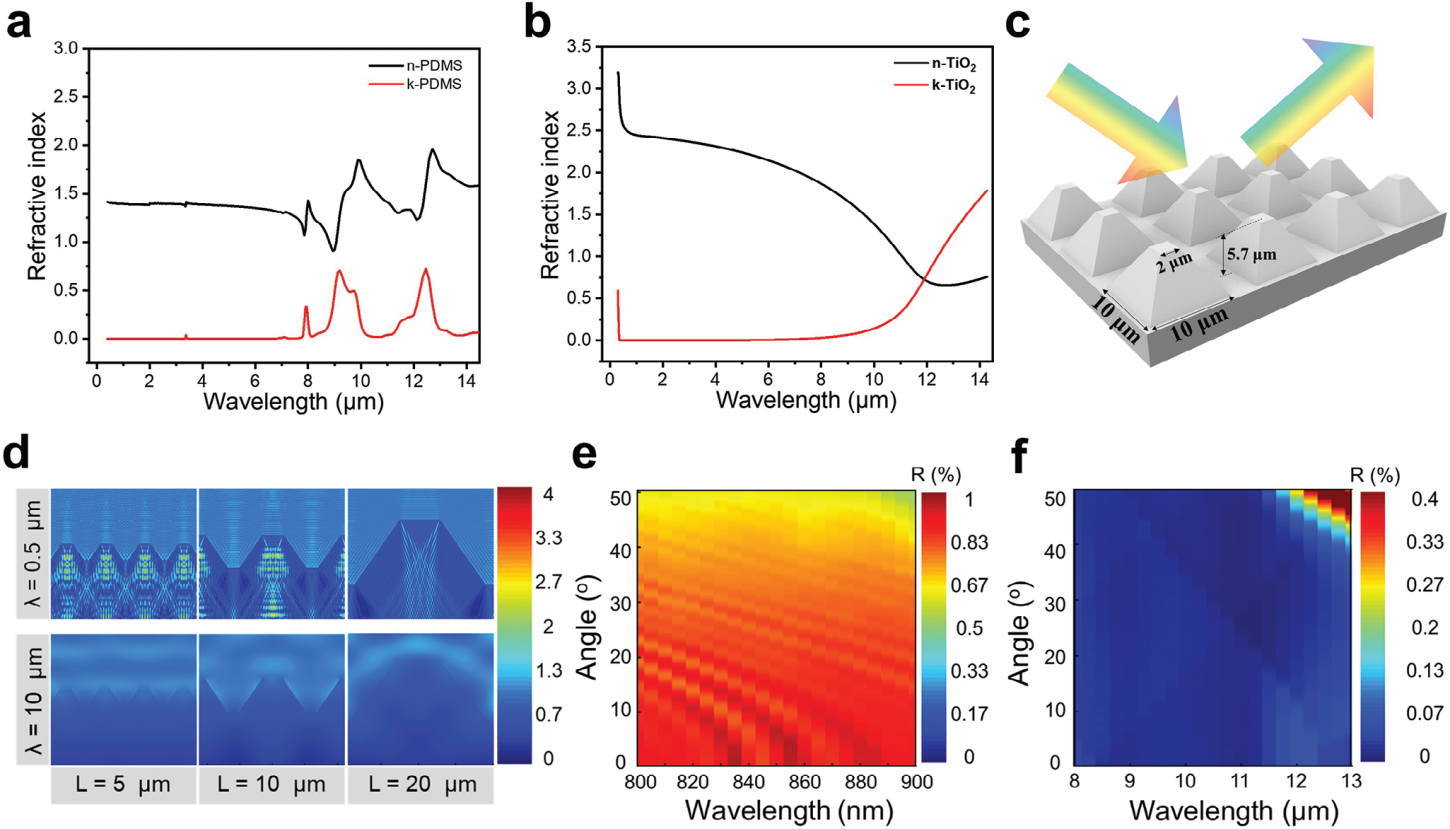
Micro‐pyramid model electromagnetic simulation and structure design of PRCM. a, b) Refractive index and extinction coefficient of the PDMS and TiO_2_. c) Typical structure of pyramidal array. d) Electromagnetic field intensity dispersion of the visible (0.5 µm) and IR (10 µm) light based on models with different pyramid sizes. e, f) Simulation of the reflectance change with varied incident light angle from 0° to 50°. Incident light wavelength, e) 0.8–0.9 µm, f) 8–13 µm.

According to the photonic crystal theory, a specific band gap can be engineered to achieve light reflection at a particular wavelength by designing materials with appropriate dielectric constant and photonic crystal size. To achieve an enhanced radiative cooling efficiency, a careful design of materials category and periodic structure is carried primarily. As a promising and low‐cost polymer, PDMS boasts a stable refractive index (n ≈ 1.4) across the vis‐NIR spectrum, while its strong functional group vibration enables its efficient heat radiation through the atmospheric window, making it an attractive material for application in radiative cooling (**Figure** **2**a). It is noting that, as a silicon rubber, PDMS possesses a strong rebound property that makes it tensile for stretching resistance. Moreover, the use of dielectric particles has been theoretically and experimentally demonstrated to be effective in advancing radiative coolers. Titanium particles (d ≈ 500 nm) were taken as an alternative to corrugated facets in the biological longicorn beetle model for their similar strong Mie scattering effect in the work (Figure 2b). Yet, the band gap of titanium dioxide determines its strong absorption in the ultraviolet region. As an anti‐ultraviolet modification layer, PVDF is critical in ultraviolet anti‐aging, especially for the 300–380 nm light. Thus, the PVDF is selected as the coating layer for ultraviolet reflection.

To determine the optimized design of periodic structure, finite‐difference time‐domain (FDTD) simulations were employed to investigate the optical properties of structures in different sizes. Inspired by the special structure of the heat‐resistant organisms, the simulated lengths of the micro‐pyramid were settled as 5, 10, 20 µm as the simulating model. Figure 2c illustrates the typical structure of a 10 µm sized pyramidal array for radiative cooling. As described in Figure 2d, the simulated area was settled as 30 µm × 20 µm, and simulated cross‐sectional dispersions of electromagnetic field intensity at a wavelength of 0.5 µm and 10 µm were mapped separately to show the optical energies distribution inside the microstructures. In the 10 µm sized model, simulated results revealed a 95% comprehensive reflection in the visible light range (0.3–2.5 µm), and only a seldom light can penetrate through the membrane. While in the atmospheric window range (8–13 µm), strong absorption is revealed intuitively. In contrast, a decreased reflection of 0.5 µm wavelength light is calculated in the 20‐µm‐sized micro‐pyramid model. This suggests that as the pyramid size increases, random media become more disordered and Maxwell‐Garnett effective medium approximation theory will no longer be adaptable in this model. As a result, the intensity of Mie scattering decreases, and the reflection in the UV‐vis/NIR region is reduced compared to that of the smaller micro‐pyramid array. As shown in Figure S2 (Supporting Information), when the simulated probe light was adjusted to 0.75 µm and 1 µm, a small amount of light penetrates the inside of the membrane, which is not conducive to radiative cooling. In the 5‐µm‐sized model, a single pyramid unit is too small for gravity deposition and multilayer TiO_2_ particles is not able to form. Stronger light intensity is detected below the structure of 5 µm sized pyramid compared to that 10 µm sized. As a result, the reflectivity decreased while emission in the MIR region also decreased as phonon polariton resonances declined. The simulating results indicated that 10 µm sized model was the most promising candidate in PRCM design.

The incidence angle can also significantly influence radiative cooling and the micro‐pyramid structure is particularly effective in enhancing total reflection. As the simulation results show in Figure 2e,f and Figure S3 (Supporting Information), strong Mie scattering occurs and the average reflectivity remains high across the visible wavelength range (0.8–0.9 µm) for incidence angles varying from 0° to 50°. When the incident light changes to wavelength range of 8–13 µm, the average reflectivity remains low for different incidence angles. According to Kirchhoff's law of thermal radiation, a decreased reflectivity corresponds to an enhanced emissivity for an unaltered transmissivity. Consequently, the broadband and wide‐angle low reflectivity contribute to high MIR emissivity for enhanced radiative cooling.

### 2.3 Preparation and Optimization of the PRCM

**Figure 3:**
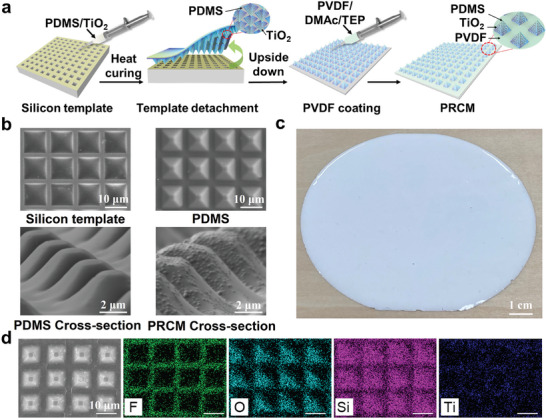
Preparation and morphology characterization of the PRCM. a) Schematic illustration of the PRCM preparation. b) SEM characterizations for PRCM synthesis. c) Physical image of PRCM. d) Energy disperse spectroscopy analysis of the PRCM. Fluorine, Oxygen, Silicon and Titanium element are detected as green, cyan, purple and blue dots respectively.

Multiple steps were involved in the manufacturing process, including photolithography, dry etching, wet etching, spin coating, and stripping from the template (**Figure** **3**a and more details in Methods part). As shown in Figure 3b, SEM images vividly show the morphology of the silicon template, pure PDMS with pyramid structure and PRCM. The pyramidal units could be observed uniformly distributed with TiO_2_ particles inside the PRCM. Due to gravity, titanium dioxide nanoparticles are enriched at the top of the pyramid, forming a concentration gradient change. The graded scattering effects result in an enhanced Mie scattering. As shown in Figure 3c, the physical image demonstrates a snowy appearance of the PRCM, which is supposed to display brilliant radiative cooling performance. Elements analysis was conducted to detect element content and distribution for every step of the PRCM preparation, as shown in Figure 3d, Figure S4 and Table S3 (Supporting Information). Silicon, Fluorine and Titanium are characteristic elements of PDMS, PVDF and TiO_2_. As the mapping results vividly show, the Fluorine element is mainly distributed in the interspace, yet a slight content can also be found above the micro‐pyramid. The results confirm that PVDF is densely coated to the gaps among micro‐pyramids without altering their morphology. The average thickness of the PVDF is verified as around 300 nm (Figure S5, Supporting Information). Oxygen and Titanium elements tend to appear above the micro‐pyramids. Silicon is found both in the interspace and pyramids. The morphology results prove the successful preparation of the gradient periodic structure. Fourier transform infrared image for characteristic peak detection further confirms the synthesis process, as shown in Figure S6 (Supporting Information). The distinct peak matched perfectly with different components of PRCM. Specially, ‐CH_2_ bond (1406 cm^−1^), Si─O─Si bond (1023 cm^−1^), and Si─CH_3_ bond (1261 cm^−1^, 806 cm^−1^) are originated from the PDMS base while ‐CF_2_ (1180 cm^−1^) togather with γ‐PVDF characteristic peak (881 cm^−1^, 510 cm^−1^) are strong confirmation of PVDF existence.

Several indexes are taken into consideration for PRCM numerical optimization. As mentioned above, the optimized size design of periodic structure was simulated through FDTD simulations as 10 µm. To verify the simulation results' accuracy and explore variables' influence on the actual test, PRCM with different sizes (5 µm, 10 µm, and 20 µm) were fabricated and characterized respectively (Figure S7, Supporting Information). As illustrated in Figure S8 (Supporting Information), the experimental results are consistent with the simulation analysis. 10‐µm‐sized PRCM exhibits a 97.3% sunlight reflectance while 96.4%, 93.8% for 5‐µm‐sized and 20‐µm‐sized, respectively. The accurate spectrum results confirm that 10‐µm‐sized PRCM is the most promising candidate for optimal radiative cooling performance. To further detect the role that special micro‐pyramid played in sunlight reflection, a shape‐memorized micro‐pyramid structure was designed. As illustrated in Figure S9 (Supporting Information), obvious micro‐pyramid collapse is detected due to external pressure, and after heating, the structure partially recovered to a rose‐like appearance. Sunlight reflection decreased from ≈96% to ≈92% and finally recovered to ≈95% after shape recovery. The shape memory recovery experiment efficiently demonstrate the reflection enhancement attributed to the micro‐pyramid structure. Besides, the mass fraction of TiO_2_ was a crucial factor in determining the reflection of solar light. As illustrated in Figure S10 (Supporting Information), low percentage doping brings in insufficient change of refractive index and thus, the reflectance of sunlight is not qualified enough for radiative cooling. Specifically, as the TiO_2_ mass fraction increased from 5% to 30%, the reflected light intensity percentages were 68.4%, 74.9%, 82.5%, 88.7%, 90.8% and 92.3%, respectively in flat samples, while a prominent reflective improvement by introducing the micro‐pyramid structure. 79.5%, 88.7%, 93.4%, 95.1%, 97.1% and 97.3% of reflection were detected in different TiO_2_ mass fraction PRCM, illustrating the reflectance enhancement of the special pyramidal structure. It is noting that prompting the mass ratio of TiO_2_ might slightly decrease the emissivity in the MIR region. As the spectrum results shown in Figure S10 and S11 (Supporting Information), emissivity decreased from around 99% to around 97.5% as the mass fraction of TiO_2_ increased. This may be attributed to the reduced surficial proportion of PDMS, which is dominant in MIR emission. After comprehensive consideration, the mass ratio of 25% was selected as the optimal condition. Membrane thickness is another factor that influences sunlight reflection. As shown in Figure S12 (Supporting Information), results intuitively illustrate that setting the film as 800 µm is a good alternative considering that relatively stable reflectance in thicker films. As mentioned above, PVDF was served as the coating layer for UV protection and the spectrum detection of the PVDF was conducted, as shown in Figure S13 (Supporting Information). The thickness of PVDF was around 300 nm. By comparing the solar light reflectivity with or without PVDF coating, the enhancement of PVDF on the reflection of 300–380 nm ultraviolet light is highlighted.

### 2.4 Spectrum Analysis and Cooling Performance Detection of PRCM

**Figure 4:**
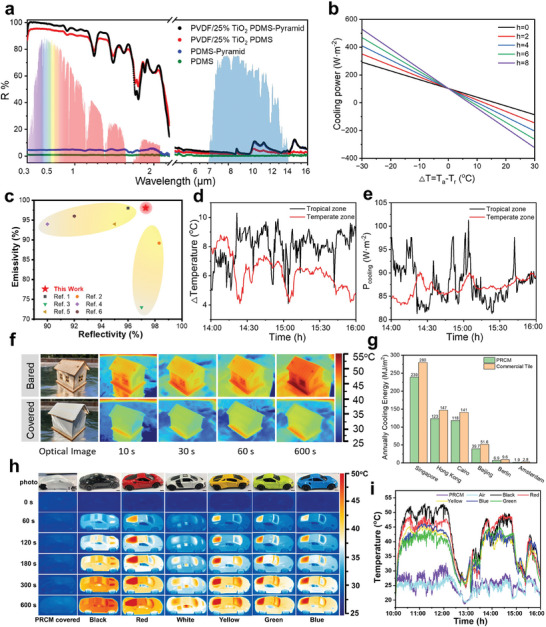
Daytime radiative cooling performance test and applications exploration of PRCM. a) Reflection spectrum detection of different membranes. Flat PDMS film (green line), PDMS film with micro‐pyramid structure (blue line), 25% TiO_2_ doped flat PDMS film with PVDF coating (red line) and PRCM (black line) were set as contrasts. Iridescence zone was drawn based on the ASTMG173 global solar spectrum, and blue area represents the atmospheric absorptivity/emissivity in the TASW. b) Calculations of the net cooling power P_net_ as a function of the temperature drop ΔT with different effective conductive‐convective heat transfer coefficients *h*. The ambient temperature is set as 30 °C. c) Solar light reflectance and MIR emissivity compared with the existing works. d) Real‐time temperature difference between PRCM and ambient temperature in actual test. e) Measured real‐time cooling power of PRCM. f) PRCM radiative cooling performance test on a cabin model. g) Simulated analysis of annual energy consumption by endowing PRCM. Six representative cities are selected, including Singapore, Hong Kong, Cairo (tropical), Beijing (Temperate), Amsterdam, and Berlin (Cold zone). h) PRCM radiative cooling performance test on the vehicle models. i) Real‐time temperature records in outdoor actual test.

As mentioned above, the bioinspired PRCM has been manufactured and optimized for radiative cooling. To quantitatively characterize its cooling performance, spectroscopic detection in the solar and MIR regions (8‐13 µm) was primarily carried out. As illustrated in **Figure** **4**a, PDMS is transparent to the UV–vis light with only 1.1% reflectance (green line). As the Fresnel equation described, light reflection enhanced as the incidence angle increased. The introduction of micro‐pyramid structure promotes the reflectance to around 4.7% as a contrast (blue line). Due to the strong Mie scattering that TiO_2_ brings to the composite membrane and enhancement of PVDF coating on the reflection of 300–380 nm ultraviolet light, a total 90.8% reflectance is exhibited, as shown in red line. However, this reflectivity is insufficient in daytime radiative cooling since a 1% power density of solar light will bring around 10 W m^−2^ to the membrane, and it is essential to elevate the reflectivity to a further step. By introducing the micro‐pyramid structure, solar light reflectivity increased to 97.1% (more details are given in the SI Appendix for solar light reflectivity calculation). As the main component of the PRCM, Si‐O‐Si, Si‐C and C─H bonds were involved in PDMS. Through the stretching and vibration of the molecule, PRCM realized strong MIR (8‐13 mm) emissivity, which indicated radiative heat through the atmospheric window. MIR spectrum results showed 96.7%, 99.3%, 98.0%, and 96.7% emissivity for pure PDMS, micro‐pyramid PDMS, doped PDMS and PRCM, respectively, in the atmospheric window range. The theoretical net cooling power (P_net_) of the PRCM can be calculated based on the heat balance equation as 80–103 W m^−2^, varied with the instant temperature and humidity (Figure 4b, more details are given in the SI Appendix). The optical performance of the PRCM has been compared with references (Table S1, Supporting Information) under the similar thickness (> 600 µm) as shown in Figure 4c. Compared to films with similar thickness, the preferable optical performance of PRCM is illustrated.

According to the analysis above, the optimized PRCM was manufactured and supposed to have a strong radiative cooling capacity for practical use. A series of practical experiments were carried out to illustrate radiative cooling performance of the PRCM. Shenzhen (114°0′17″E, 22°36′29″N) and Qingdao (120°31′28″E, 36°10′01″N) were represented as the tropical and temperate climate cities respectively to carry the experiments in late June. A homemade device was settled for the test, as shown in Figure S14 (Supporting Information). The influence of thermal convection and conduction was strictly controlled in the experiment for their negative influence on the cooling performance of the PRCM. In this way, a styrofoam box wrapped by thermal insulation materials was used to load the PRCM and a polyethylene film was sealed closely above the box for isolating convection. The PRCM placed in the device was subjected to direct sunlight under a clear sky. Temperatures were detected during the hottest period of the day and the corresponding temperature difference (ΔT) was calculated in Figure 4d. Humidity and solar intensity were also recorded in real‐time as shown in Figures S15,S16 (Supporting Information). In Shenzhen, the average solar intensity I_solar_ was ≈778 W·m^−2^ with an average 65% relative humidity at noon. An average temperature difference of 7.3 °C was monitored on the PRCM while the instantaneous value reached 10.3 °C. While in Qingdao, an average temperature difference of 6.1 °C with an instantaneous maximum ΔT of 9.7 °C was detected. The superior cooling performance in Shenzhen was ascribed to the higher ambient temperature in the test, which benefited MIR emissivity. To quantify the measurements, we further analyzed the experimental cooling performance in a home‐made device by electrical heating to compensate for the temperature drop (Figure S17, Supporting Information). The average daytime P_net_ of the film was obtained as 93.5 W·m^−2^ and 87.3 W·m^−2^ in Shenzhen and Qingdao respectively (Figure 4e). In theory, the ideal P_net_ of the PRCM calculated by theoretical equations (SI Appendix) is around 103 W·m^−2^, considering a 37 °C (310 K) ambient temperature without any thermal convection or conduction. The slight decrement value of P_net_ could be ascribed to the unavoidable thermal convection and conduction. The PRCM is supposed to have various application scenarios for its brilliant cooling performance. A house model was applied to test the radiative cooling performance of the PRCM for building thermal management and the results are shown in Figure 4f. By covering PRCM closely, a decrement of up to 7 °C was observed after 600 seconds of sunlight exposure. This experiment directly varied the promising future of our materials in building radiative cooling. An energy consumption simulation was carried out based on the model of PRCM. As illustrated in Figure 4g, six cities were chosen as the simulating places, including three in tropical zone (Singapore, Hong Kong and Cairo), one in temperate zone (Beijing) and two in cold zone (Amsterdam and Berlin, more details are given in the SI Appendix and Figure S18 (Supporting Information) for energy consumption simulation). Energy consumption was calculated mainly based on building cooling for proper inside temperature, and air‐conditional devices were set to work only at hyperthermal (beyond 30°C). The simulation results were consistent with our common sense that extreme heat brought more energy consumption especially from June to August in tropical regions. The bared houses tended to call for more annual energy consumption in hotter areas, and around 15% of energy saving was obtained by the PRCM. Specifically, after endowing with the PRCM, annual energy consumption declined from 280 MJ m^−2^, 147 MJ m^−2^, and 141 MJ m^−2^ to 239 MJ m^−2^, 123 MJ m^−2^, and 118 MJ m^−2^ in Singapore, Hong Kong and Cairo, respectively. Similar result was drawn from temperate cities that annual energy consumption in Beijing declined from 52 MJ/m^2^ to 40 MJ/m^2^ by covering the PRCM. Under the current stage, building cooling is not common in cold zone cities as the temperature there tends to be pleasant, yet 2.7 MJ m^−2^ and 0.9 MJ m^−2^ annually energy consumption decrement was still found in the simulation results of Berlin and Amsterdam. In addition to excellent optical performance, the PRCM also demonstrated superior hydrophobic properties, making it feasible for building coverage (Figure S19, Supporting Information). After 5 minutes of running water rush, most of the dust would be removed from the PRCM, as shown in the dust resistance experiment (Figure S20, Supporting Information). Considering the actual outdoor application scenario of PRCM, rainwater can serve as a straightforward way to protect the PRCM from dust disturbance in the long‐term use. Besides, thanks to the strong ductility of the silicone rubber, the loading stress is almost unchanged after 10000 cycles of stretching, indicating that the mechanical properties of PRCM are almost unaffected (Figure S21, Supporting Information). The excellent flexibility and elasticity endow the PRCM with longer durability and broader applications. It is noting that the PRCM preparation is cost‐effective at around $82.8 per square meter (Table S4, Supporting Information), which lays the foundation for its wide applications. As another common practical scenario, vehicle models were also selected as the experiment object for the PRCM. As shown in Figure 4h, after receiving 600 seconds of illumination from the solar light simulator, a 11.6°C to 22.5°C increment was detected on the car models while only 1.8°C increment was observed with PRCM coated. Compared with commonly used commercial car clothes with similar thickness, the PRCM performs a preferable optical performance (Figure S22 and Table S5, Supporting Information), which paves the way for practical application. Ourdoor real‐time test illustrated a similar result as shown in Figure 4i and Figure S23 (Supporting Information). Around 10 °C temperature difference was recorded between the PRCM fixed and those uncovered vehicles when exposed to the sun, which is effective to show the radiative cooling ability of the PRCM.

## 3 Conclusion

In summary, a bioinspired PRCM is prepared by integrating high dielectric constant materials with polymers for building thermal management and outdoor practical scenarios. Both direct detections and simulations revealed the enhanced reflectance of solar light and increased emissivity of MIR. Moreover, based on the practical daytime radiative cooling experiment, the PRCM exhibited a superior radiative cooling performance, achieving an average temperature drop of 7.3 °C for the coated device. These findings shed light on passive radiative cooling for cooling buildings. Furthermore, considering the simplicity of the preparation and the ecological efficiency of this strategy, it holds great promise for pioneering a new approach to thermal management across diverse natural environments.

## 4 Experimental Section

### Chemicals

75‐nm‐thick Silicon Nitride wafers were bought from Suzhou Silicon Electronic Technology Co., LTD. Titanium oxide (anatase) and polyvinylidene fluoride (PVDF) were bought from Shanghai Aladdin Biochemical Technology Co., Ltd. Polydimethylsiloxane (PDMS, SYLGARD™ 184 silicone elastomer base) was bought from Dow Corning (China) Silicone Co., LTD. m‐Xylylenediamine (MXDA), Epoxide resin (E‐51) and n‐Octylamine were bought from Shanghai Macklin Biochemical Co., Ltd. Deionized water was obtained by a Milli‐Q water‐purification system. All commercially available chemicals were used without further purification unless otherwise noted.

### Characterizations

Original optical images of the bioinspired radiative cooling films were photographed by a smartphone without further treatment. Infrared images were obtained from the infrared thermal imager (Fluke, Ti480U) and results were visualized in the SmartView software. In addition, thermocouple thermometer (TASi, TA612) was used to reveal real‐time temperature variation of the PRCM. A field emission scanning electron microscope (FE‐SEM, Hitachi SU8230) was used to observe microstructures. Meanwhile, elementary composition was detected from an energy dispersive X‐ray spectrometer (EDS, Bruker Quantax Wds). The solar reflectivity of PRCM was tested on a UV–Vis/NIR spectrophotometer with an integrating sphere (Shimadzu ISR‐1503). The infrared transmittance spectrum was obtained from the Fourier Transform Infrared (FT‐IR) spectrometer. MIR spectrum detection was carried on an infrared spectrometer (Bruker Vertex 70v) equipped with mercury cadmium telluride (MCT) detector and MIR integrating sphere. Refractive index of TiO_2_, PVDF and PDMS were adopted from the website (https://www.refractiveindex.info./). Cyclic tensile stability measurement of the PRCM was carried out at room temperature using a dynamic thermo‐mechanical analyzer (DMA, Instron 68TM‐30). The contact angles of water/air droplet were measured by drop shape analyzer (SDC‐200S). Purified water (10 ill, with a surface tension of 72.0–73.0 Mn m^−1^) was used as the model liquid. Full‐wave simulations were conducted by FDTD Solutions (V8.21.1882, Lumbricals Could) to simulate solar light reflection and emission in the atmospheric window. For the Bio‐RC films, the refractive index data was obtained from the refractive index database.

### Biological Sample Processing

The silver ants (*C. Bombycina*) were bought from Chinese electronic markets and longicorn beetle specimens (*A. germari*) were provided by the key laboratory of forest protection of national forestry and grassland administration, ecology and nature conservation institute, Chinese academy of forestry. The specimens were soaked with 10% formalin solution for over 6 hours to fix biological microstructure. The forewings of the longicorn beetles were cut off using a sharp blade for better investigation. The obtained forewings were rinsed with ethyl alcohol and water and received an ultrasound process to remove impurities attached to the surface. For pigment removal, the forewings were completely immersed in a 10% hydrogen peroxide (H_2_O_2_) solution for 48 h and then rinsed thoroughly with purified water.

### Synthesis of PRCM

Three steps were included in the PRCM preparation. In the first step, silicon wafer templates with different (5, 10, 20 µm) inverse‐pyramid array were obtained through photolithography, dry etching, wet etching, spin coating, and stripping. Specifically, photolithography and reactive ion etching were applied to form a square array on the silicon nitride layer at the top of a (100) silicon wafer. Then, the patterned silicon nitride layer was served as a photomask and anisotropic etching was performed with potassium hydroxide (KOH) to obtain silicon templates with periodically inverted micro‐pyramid on the surface. In the next step, the titanium dioxide (TiO_2_) particles were uniformly mixed into the PDMS prepolymer to form a precursor solution. The mass ratio of the TiO_2_ was varied from 5% to 30%. Subsequently, precursor solution was added to the template and received a spin coating. After thermal curing at 80°C for an hour, a pyramid‐shaped composite film was formed. Finally, after receiving a plasma process, hydroxyl groups were produced on the surface of the composite film for an adhesive surface. 10 wt.% Polyvinylidene fluoride (PVDF) particles were dissolved in the dimethylacetamide (DMAc)/ triethyl phosphate (TEP) solution (mass ratio = 3:2) and added to the composite film. By placing at a humid environment, PVDF was precipitated out and formed a UV reflection coating for the micro‐pyramid.

### Synthesis of Reversible Micro‐Pyramid Structure Membrane

The prepolymer of shape memory polymer was composed of A diglycidyl ether, n‐octylamine, and m‐xylylenediamine, which was mixed up in a molar ratio of 4:2:1 (weight ratio 8: 2.54: 1.36). By degassing for 10 min, the SMP prepolymer mixture was then poured onto the silicon template. After being kept at 60 °C for 2 h and baked at 100 °C for an hour in sequence, the superhydrophobic SMP was finally obtained after carefully peeling from the silicon template.

## Conflict of Interest

The authors declare no conflict of interest.

## Author Contributions

Q.‐H.P. and M.‐H.W. contributed equally to this work. Z.H. and S.‐H.Y. conceived the idea and designed the experiments. Z.H., J.‐L.W. and S.‐H.Y. supervised the research. Q.‐H.P., X.‐J.Q., M.‐H.W., X.G., and C.C. carried out the experiments and characterizations. M.‐H.W. performed the simulation analyses. Y.‐T.W. carried out the FIB testing. M.‐H.Z. and F.‐X.Z. characterized the SEM and EDS analysis. Z.‐Y.H. drew the diagram of the manuscript. S.‐C.Z. prepared the silicon template. Q.‐H.P. summarized the data and wrote the manuscript. All authors participated in results analyzation and discussion.

## Supporting information



Supporting Information

## Data Availability

The data that support the findings of this study are available in the supplementary material of this article.
