# Supplementary material for: Biomimetic Pyramid Structure Film for Enhancing Building Radiative Cooling
Source: Adv Sci (Weinh). 2025 Feb 14;12(14):2413559. doi: 10.1002/advs.202413559 (PMC11984879; doi:10.1002/advs.202413559)
Supplement: Supplementary file 1 — Supporting Information [file ADVS-12-2413559-s001.docx]

**Biomimetic Pyramid Structure Film for Enhancing Building Radiative Cooling**

*Qian-Hao Pan^#^, Mei-Hua Wang^#^, Zong-Ying Huang, Xiao-Jing Qiu, Yu-Tao Wang, Fu-Xing Zhao, Meng-Han Zhu, Xin Guo, Chen Chen, Si-Chao Zhang, Jin-Long Wang, Zhen He*, and Shu-Hong Yu**

Q. H. Pan, M. H. Wang, Z. Y. Huang, X. J. Qiu, Y. T. Huang, F. X. Zhao, M. H. Zhu, X. Guo, C. Chen, Prof. J. L. Wang, Prof. Z. He*, and Prof. S. H. Yu*

Shenzhen Key Laboratory of Sustainable Biomimetic Materials

Guangdong Provincial Key Laboratory of Sustainable Biomimetic Materials and Green Energy

Department of Materials Science and Engineering

Institute of Innovative Materials

Guangming Advanced Research Institute

Southern University of Science and Technology

Shenzhen 518055, China.

E-mail: hez@sustech.edu.cn; [yush@sustech.edu.cn](mailto:yush@sustech.edu.cn)

S. C. Zhang,

Department of Chemistry

University of Science and Technology of China

Hefei 230026, China.

Prof. S. H. Yu

New Cornerstone Science Laboratory

Division of Nanomaterials & Chemistry Hefei National Research Center for Physical Sciences at the Microscale

Department of Chemistry

Institute of Biomimetic Materials & Chemistry

Anhui Engineering Laboratory of Biomimetic Materials

University of Science and Technology of China

Hefei 230026, China.

#These authors contributed equally to this work.

**I.**  **Supplementary Text**

1. Finite-different time-domain (FDTD) analysis for mechanism interpretation.

2. Calculations of refractive index.

3. Simulated energy consumption of buildings.

4. Calculated radiation cooling power of the Bio-RC film.

**II. Supporting Figures.**

Figures S1 to S22

**III. Supporting Tables.**

Table S1 to S4

**1. Finite-different time-domain (FDTD) analysis.**

To determine the optimized design of periodic structure, FDTD simulations was conducted. The simulation models were built identically to the actual micro-structure of the PRCM. Materials used in the simulation consisted of 25 wt.% TiO_2_ doped PDMS membrane, with a 300-nm-thick PVDF layer coated on the surface. A planar light source served as the simulating far-field solar light source. The inside and surface light energy flow of the micro-structured model were investigated respectively. The simulation included micro-pyramid structure with varied sizes, such as 5 μm, 10 μm, 20 μm and the simulated cross-section electromagnetic field intensity dispersion diagrams were presented as Figure 2d. To assess the impact of different incident angles on the reflectivity, simulations were carried out with incident angle ranging from 0^o^ to 50^o^. The simulated reflectivity diagram of the micro-pyramid model at these varing incident angles were presented for the wavelength range of the 0.8-0.9 μm and 8-13 μm respectively (Figure 2e, f). The results of the FDTD simulations further validate the effectiveness of micro-pyramid structures for enhancing the radiative cooling performance.

**2. Calculations of refractive index.**

According to the Maxwell-Garnett effective medium approximation, the dielectric function for doped PDMS matrix is determined as follows:

$\frac{\varepsilon_{eff}-\varepsilon_{n}}{\varepsilon_{eff}+2\varepsilon_{n}}=f\frac{\varepsilon_{n}-\varepsilon_{h}}{\varepsilon_{n}+2\varepsilon_{h}}$ (1)

where $\varepsilon_{eff}$, $\varepsilon_{n}$, and $\varepsilon_{h}$ are the dielectric functions of matrix and particles, 𝑓 is the volume fraction of particles. The conversions of dielectric constant to refractive index (n) are as follows:

${\varepsilon_{n}(\lambda)}=n^{2}$ (2)

**3. Simulated energy consumption of buildings.**

Simulations on EnergyPlus was carried out to assess the energy-saving performance of PRCM. The 5×4×3 m^3^ house model came from the practical factory, featuring with three 1.6×1.2 m^2^ and two 1.5×1.5 m^2^ windows (Figure S17). PRCM and commercial tile were applied as the outside coating layer of all the external walls and roofs simultaneously. The optical properties and simulating parameters are listed in Table S2. Singapore, Hong Kong, Cairo, Beijing, Berlin, and Amsterdam were selected as the representative for different types of climate zones worldwide. In the energy consumption simulations, the heating, ventilation and air conditioning (HVAC) system was set to activate only when the indoor temperature exceeded 30°C, and the cooling equilibrium temperature point was settled as 26℃. The results in the Figure 4g show the annual energy consumption of the six cities. The total energy consumption of the house is significantly reduced when equipped with PRCM compared to the bared commercial tile, especially during the hot summer. Moreover, the house energy-saving performance was quantified by measuring the differences in total annual energy consumption between PRCM and commercial tile. The simulations indicate that total energy consumption for cooling would be promisingly reduced by 14.9%, 16.4%, 16.3%, 23.2%, 28.4%, and 30.1% in Singapore, Hong Kong, Cairo, Beijing, Berlin, and Amsterdam, respectively. Thus, applying PRCM would shed light on energy consumption saving while reducing carbon emissions for a friendly environment.

**4. Calculated radiation cooling power of the Bio-RC film.**

The energy balance between the PRCM and surronding environment is illustrated in Figure 3c. Considering the co-existance of heat conduction, heat convection and heat radiation. The net cooling power (P_net_) can be calculated as the follows:

$P_{net} = P_{rad}(T_{r})-P_{sky}-P_{solar}-P_{cond-conv}$ (1)

In this equation, *T_r_* represents the temperature of radiation since radaition intensity is closely determined by the temperature. Considering the actual performance of PRCM, the thermal radiation power *P_rad_* can be calculated by:

$P_{rad}=\int_{0}^{+\infty} \int_{0}^{2\pi} \int_{0}^{\frac{\pi}{2}} \varepsilon_{r}\left( \lambda, \theta,\varphi,T_{r} \right)I_{b}\left( \lambda,T_{r} \right)\cos\left( \theta\right)\sin\left( \theta\right)d\theta d\varphi d\lambda$ (2)

where$\varepsilon_{r}\left( \lambda, \theta,\varphi,T_{r} \right)$ represents the spectral emissivity in the ATSW, $T_{r}$ represents the surface temperature of the PRCM, $\varepsilon_{r}\left( \lambda, \theta,\varphi,T_{r} \right)$ represents the special emissivity and $I_{b}\left( \lambda,T_{r} \right)\cos\left( \theta\right)$is the intensity of black-body radiation at environmental temperature. According to the Planck equation:

$I_{b}\left( \lambda,T_{r} \right)\cos\left( \theta\right)=\frac{2hc^{2}}{\lambda^{5}}\frac{1}{e^{\frac{hc}{\lambda kT}-1}}$ (3)

Where *h*, *c* and *k* represent Planck’s constant, velocity of light invacuum and Bolzmann’s constant respectively. As the experiment carried in the summer around the Tropic of Cancer and thus, the azimuth angle $\varphi$ can be neglected. Eqution can be simplified as follows:

$P_{rad}=\pi\int_{0}^{+\infty} \int_{0}^{\frac{\pi}{2}} \varepsilon_{r}\left( \lambda, \theta\right)I_{b}\left( \lambda,T_{r} \right)\sin\left( 2\theta\right)d\theta d\lambda$ (4)

According to the Kirchhoff’s law of thermal radiation, the spectral absorbility of the PRCM equals to the spectral directional emissivity $\varepsilon_{r}\left( \lambda, \theta\right)$. Thus, the spectral directional radiation power of the sky atmosphere can be sdescribed as folllows:

$I_{S}\left( \lambda, \theta,T \right)=\varepsilon_{S}\left( \lambda, \theta\right)I_{b}\left( \lambda,T_{a} \right)$ (5)

where $\varepsilon_{S}\left( \lambda, \theta\right)$ refers to the spectral direcrional emissivity of the sky atmosphere, which can be expressed as:

$\varepsilon_{S}(\lambda, \theta) = 1 - {[\tau_{s}(\lambda, 0)]}^{1/cos (\theta)}$ (6)

where $\tau_{s}(\lambda, 0)$ represents the atmospheric transmissivity at verticle direction. T_a_ is the ambient temperature.

The absorbed solar radiation can be defined as $P_{solar}$, which can be expressed as follows:

$P_{solar}=\int_{0}^{+\infty} \alpha_{r}\left( \lambda, \theta_{sun} \right)I_{AM1.5}\left( \lambda\right)d\lambda$ (7)

Where $\theta_{sun}$ is the incident angle of the solar light irradiating on the PRCM. $I_{AM1.5}\left( \lambda\right)$ is the AM 1.5 spectrum distribution of the radiation intensity changing with the wavelength.

The non-radiative parasitic losses $P_{cond-conv}$ are quantified by the effective conductive-convective heat transfer coefficient ℎ which can be described as follows:

$P_{cond-conv}=hA_{r}(T_{a}-T_{r})$ (8)

The average vis-NIR reflectivity of PRCM is around 96.3% and radiates 97% of the MIR heat simultanously. Not considering the influence of the non-radiative parastic losses, the net radiative cooling power $P_{net}$ gradually increases as the radiation temperature goes up with the ambient temperature fixed at 30 °C. At the same time, the maximum temperature drop of ∆𝑇 goes up with the increase of the ambient temperature. Through the theorectical prediction, the value of $P_{net}$ varying with different radiation and ambient temperatures. A significantly higher net cooling power is achieved with the decrease of the ambient temperature and the increase of the radiation temperature. Taking the non-radiative parasitic losses $P_{cond-conv}$ into consideration, when ∆𝑇 is fixed, the net radiative cooling power is dropped with the increase of the effective conductive-convective heat transfer coefficients *h*.

**Supporting Figures**


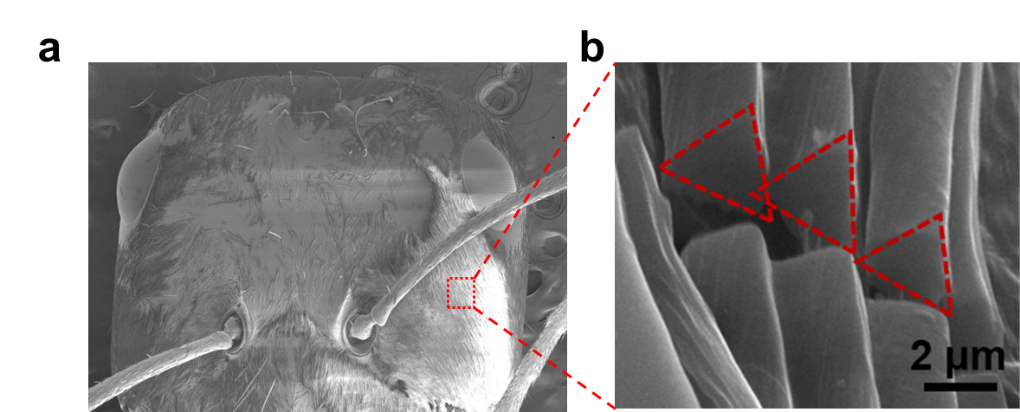


**Figure S1**│**Morphology detect of *C. Bombycina***. a) SEM detection of *C. bombycina* and b) Cross-sectional view of the hairs milled with focused ion beam (FIB).


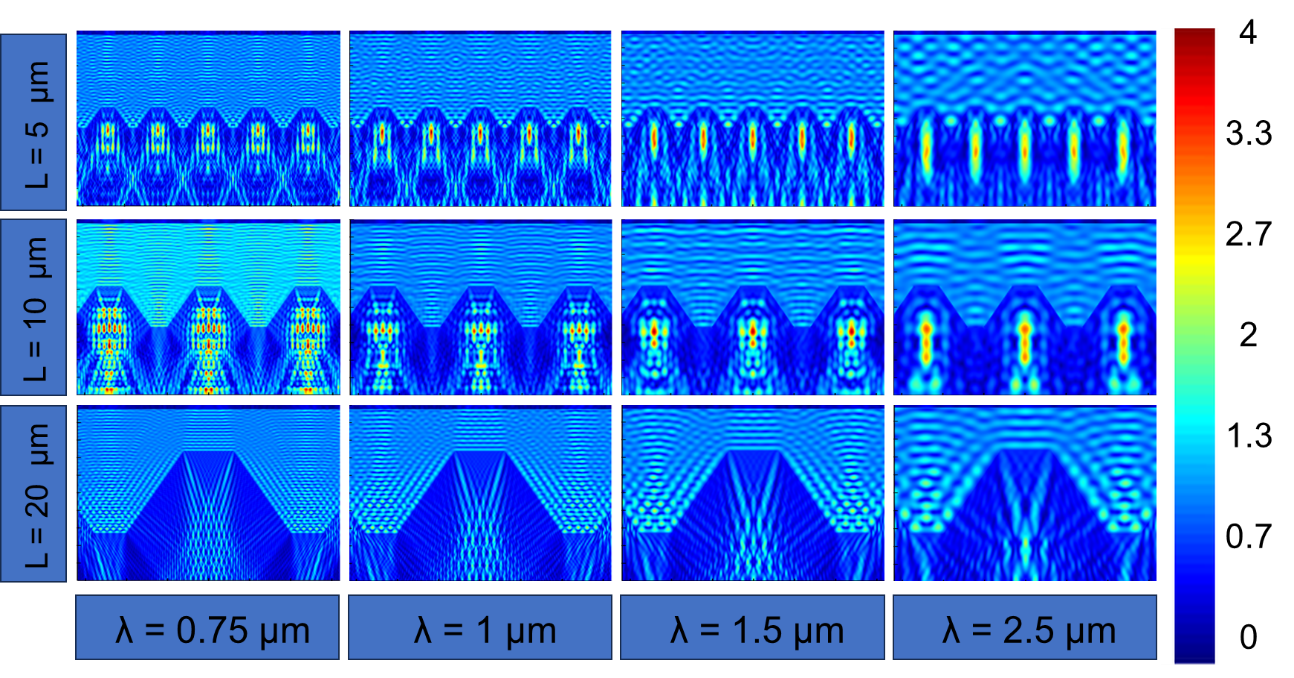


**Figure S2**│**Simulated electromagnetic field intensity dispersion diagrams of the visible and IR light of different sized micro-pyramid structures (0.75 μm, 1 μm, 1.5 μm, 2.5 μm)**.

**Figure S3**│**Simulated average reflectivity of the PRCM with different incidence angles in the 8 to 13 μm wavelength range.**


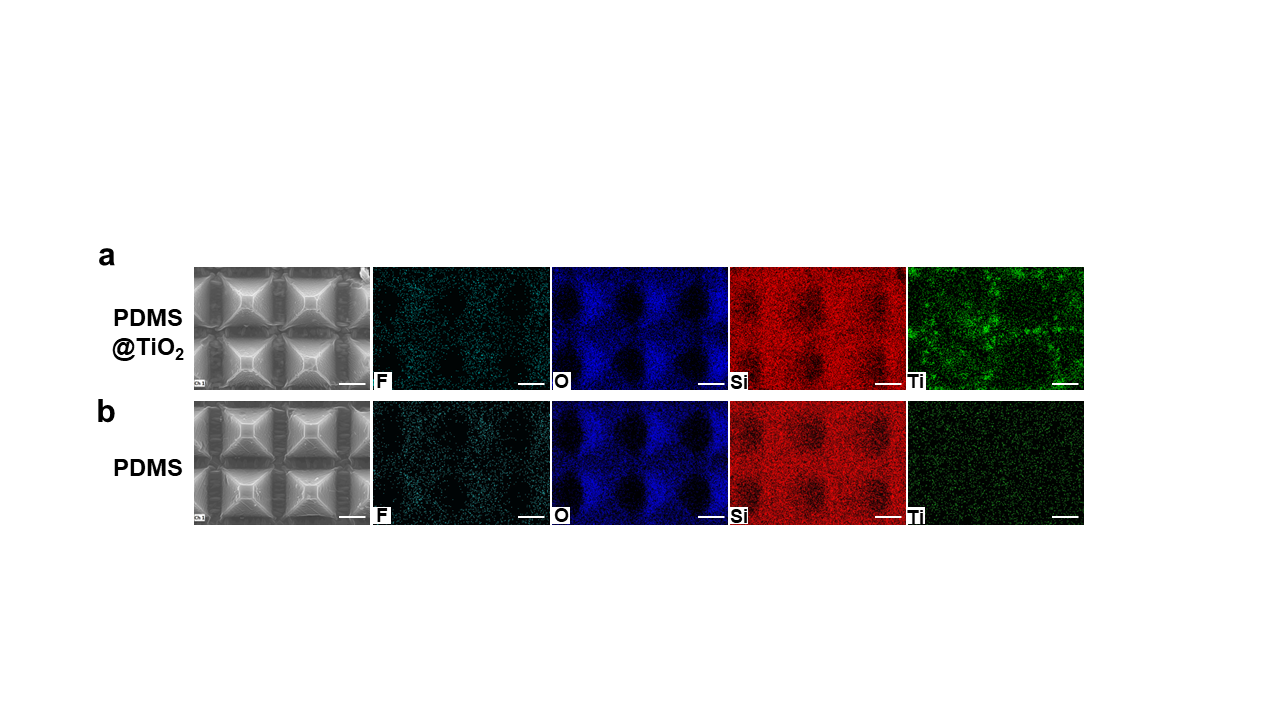


**Figure S4**│**Morphology characterization and element analysis.** SEM images illustrated the distuibution of TiO_2_ nanoparticles on the PDMS base. TiO_2_ particles are equably distuibuted with PDMS. Florine, Oxygen, Silicon and Titanium are coloured with dark green, blue, red and oliver respectively. Scale bar here is 5 μm.


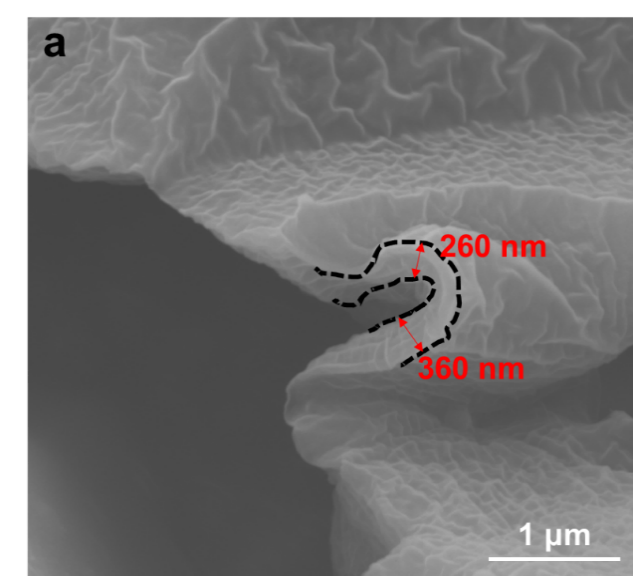


**Figure S5│Cross-section SEM images of PVDF coating.**


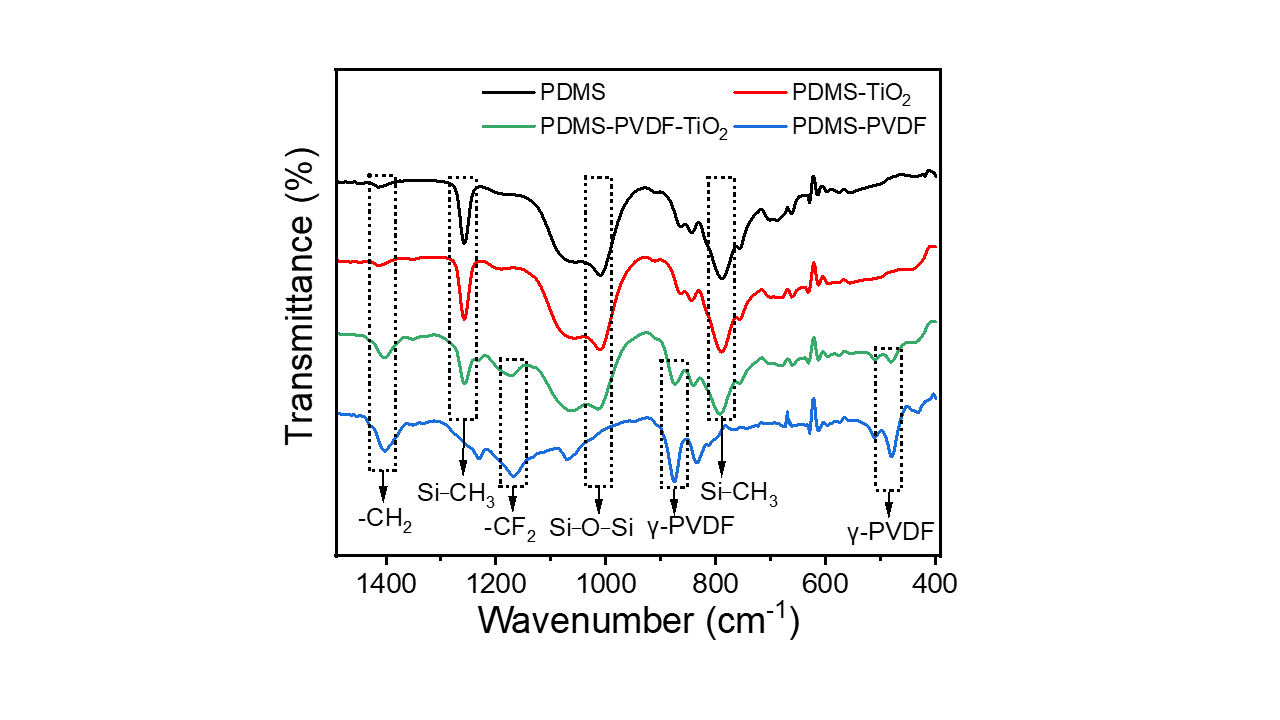


**Figure S6**│**Fourier transform infrared image for characteristic peak detection.** The characteristic peak matched different components of PRCM. Specially, -CH_2_: 1406 cm^-1^, Si-O-Si: 1023 cm^-1^ and Si-CH_3_: 1261 cm^-1^, 806 cm^-1^ are originated from the PDMS base. -CF_2_: 1180 cm^-1^,γ-PVDF: 881 cm^-1^, 510 cm^-1^ are strong confirmation of PVDF existance.


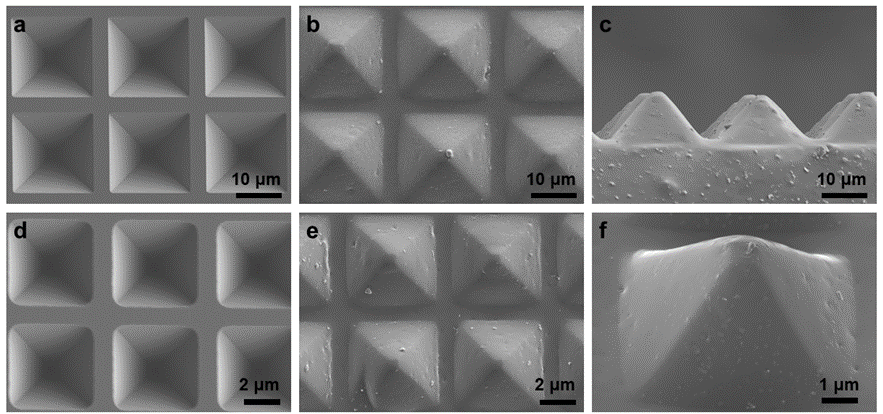


**Figure S7**│**Morphology characterization of different sized micro-pyramids** (**a-c**, 5-μm-sized PRCM, **d-f**, 20-μm-sized PRCM). Silicon template for producing 5-μm-sized PRCM (a) and 20-μm-sized PRCM (d). PDMS-TiO_2_ membrane with 5-μm-sized (b) and 20-μm-sized (e). Crossview of 5-μm-sized (c) and 20-μm-sized PRCM (f).


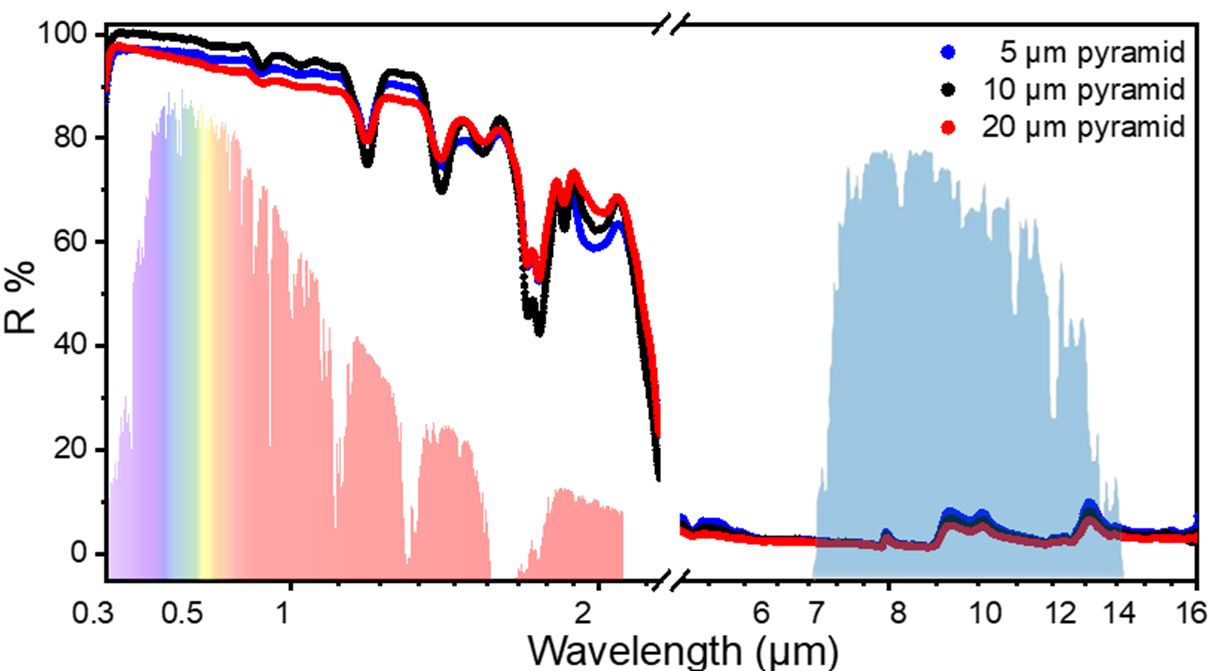


**Figure S8**│**Reflectivity detect of membranes with different pyramidal size.**


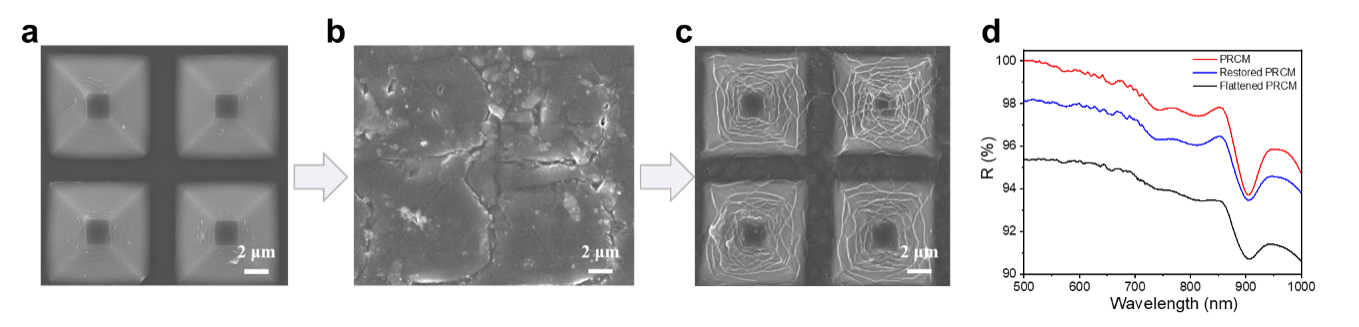


**Figure S9**│**Characterization of micro-pyramids before and after reversible shape memory experiment.** **a,** Original shape of PRCM. **b,** Structure destoyed morphology. **c,** Revisible shape of PRCM. **d,** spectrum detect (500 nm-1000 nm).


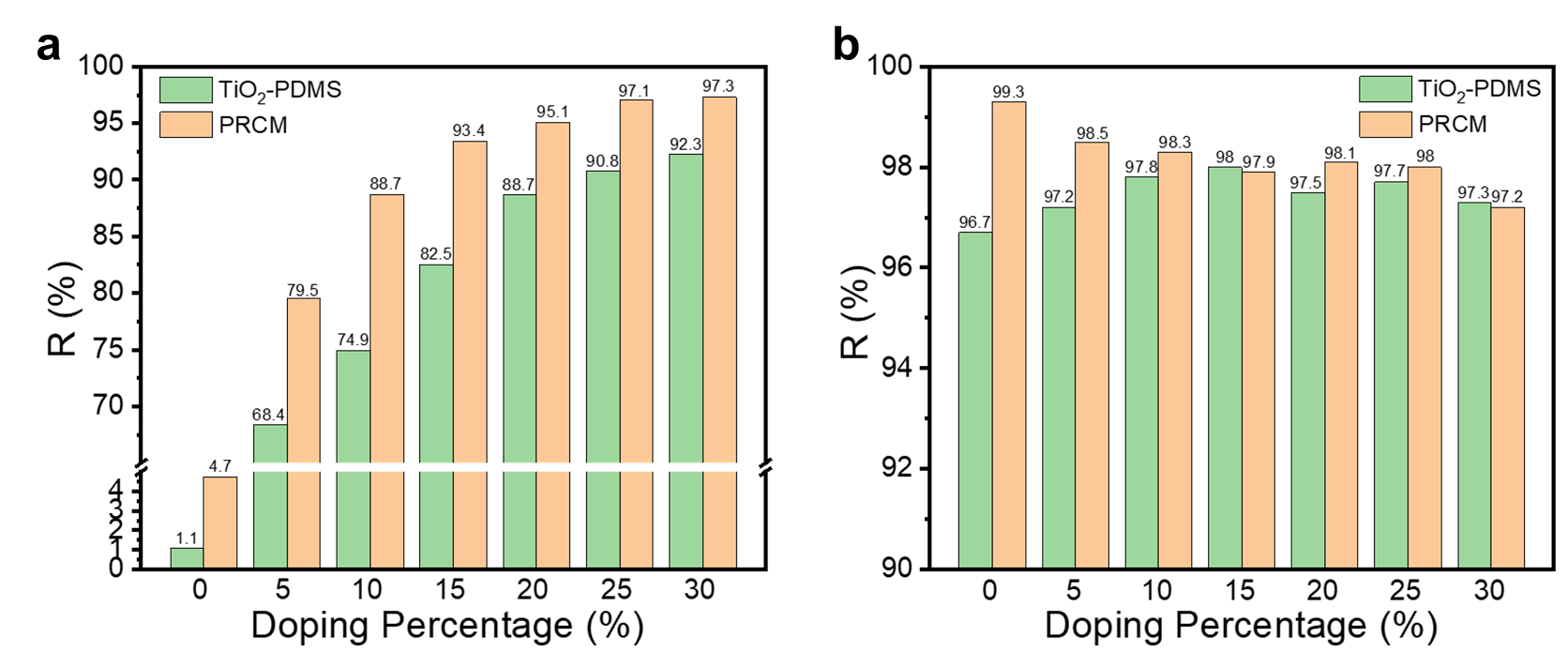


**Figure S10**│**Reflectivity detect of membranes with different doping percentage. a,** Solar light reflectance, 0.3-2.5 μm. **b,** MIR emissivity, 8-13 μm.


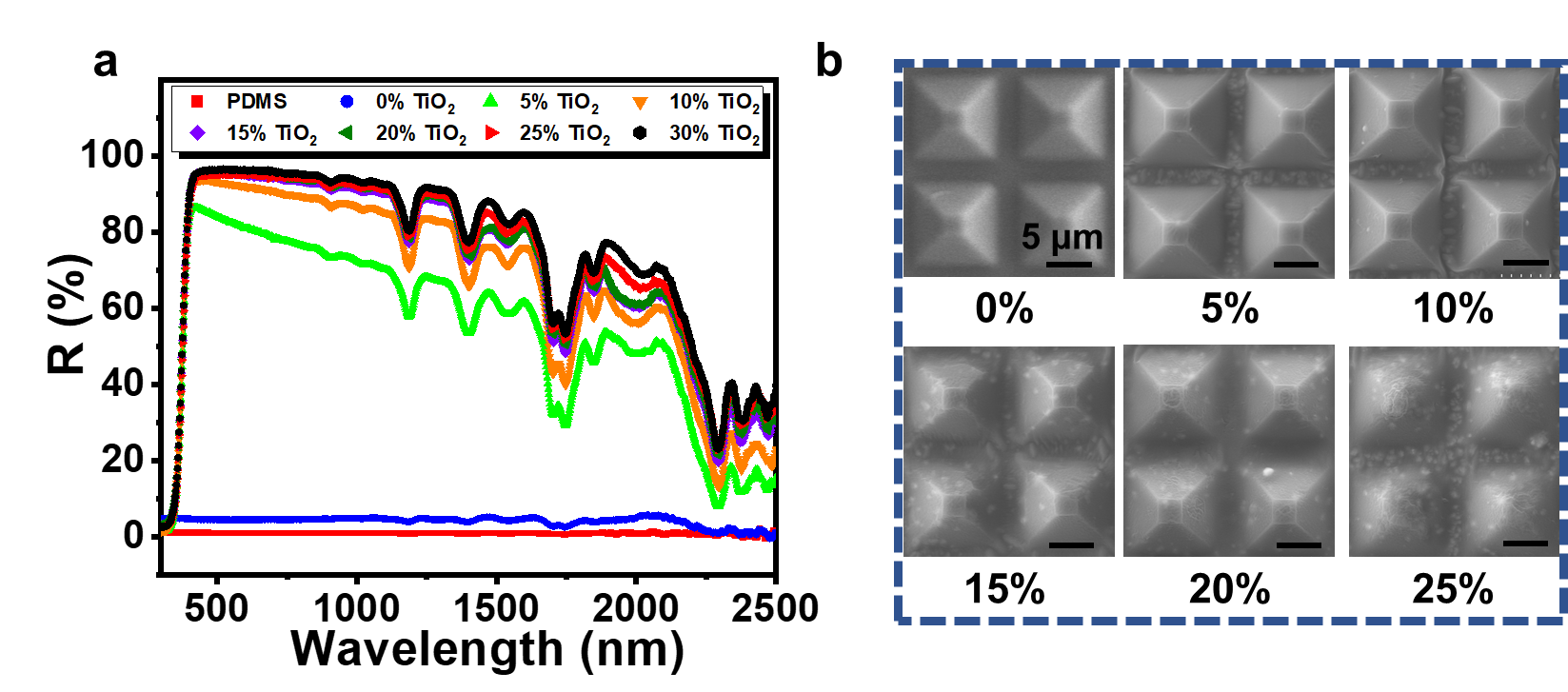


**Figure S11**│**Spectrum detection and morphology characterization of different TiO_2_ doped micro-pyramid membranes. a,** Reflection detect of different TiO_2_ doped micro-pyramid membranes with detect wavelrngth from 0.3 μm to 2.5 μm. **b,** SEM images of different TiO_2_ doped micro-pyramid membranes.


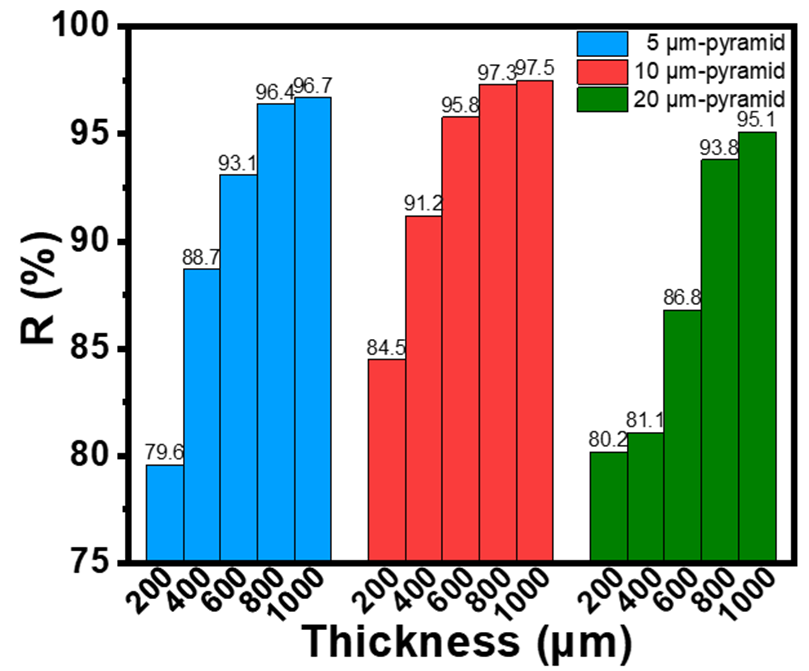


**Figure S12**│**Reflectivity detect of membranes with different thickness.**

**
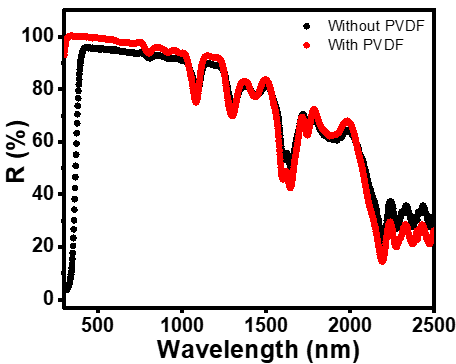
**

**Figure S13**│**Reflectance detect of membranes with and without PVDF coating.**


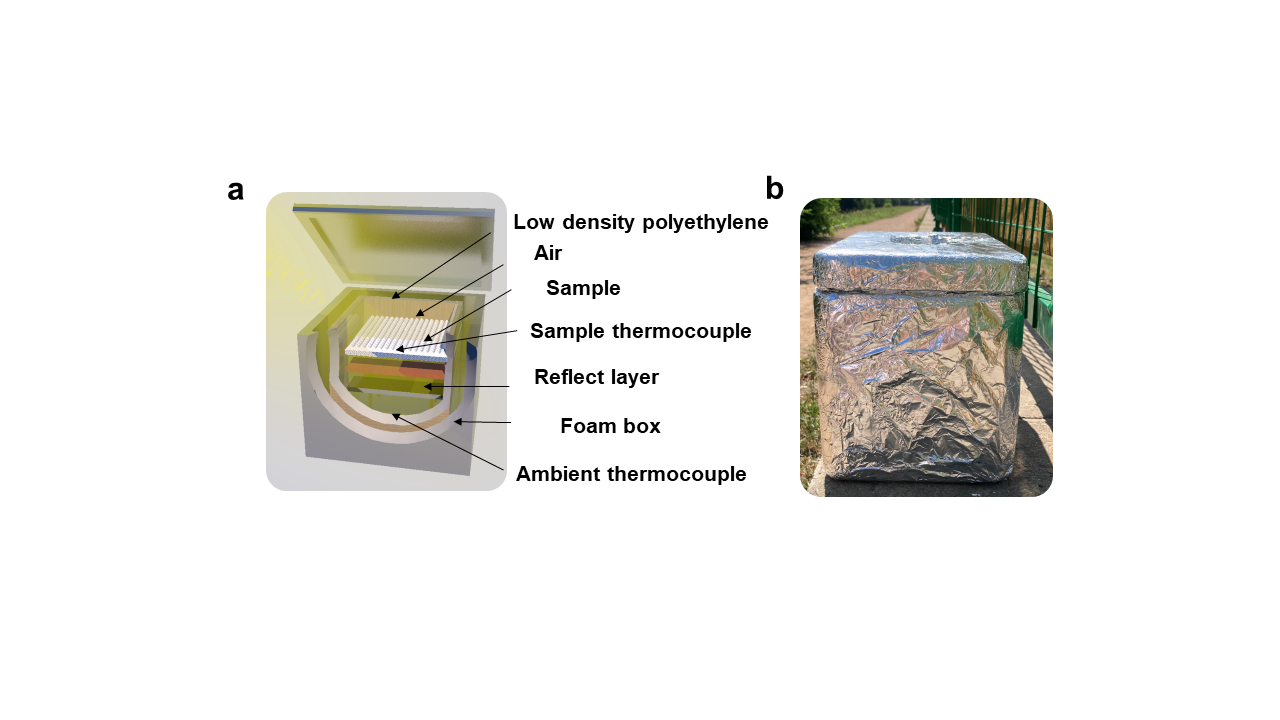


**Figure S14**│**Schematic and digital images of home-made outdoor radiative cooling test device.** **a,** Schematic image, from the top to the bottom are low density PE, sample, reflect layer. The device is loaded in a foam box for thermal isolation. **b,** digital image of the test device, setting half a meter off ground.


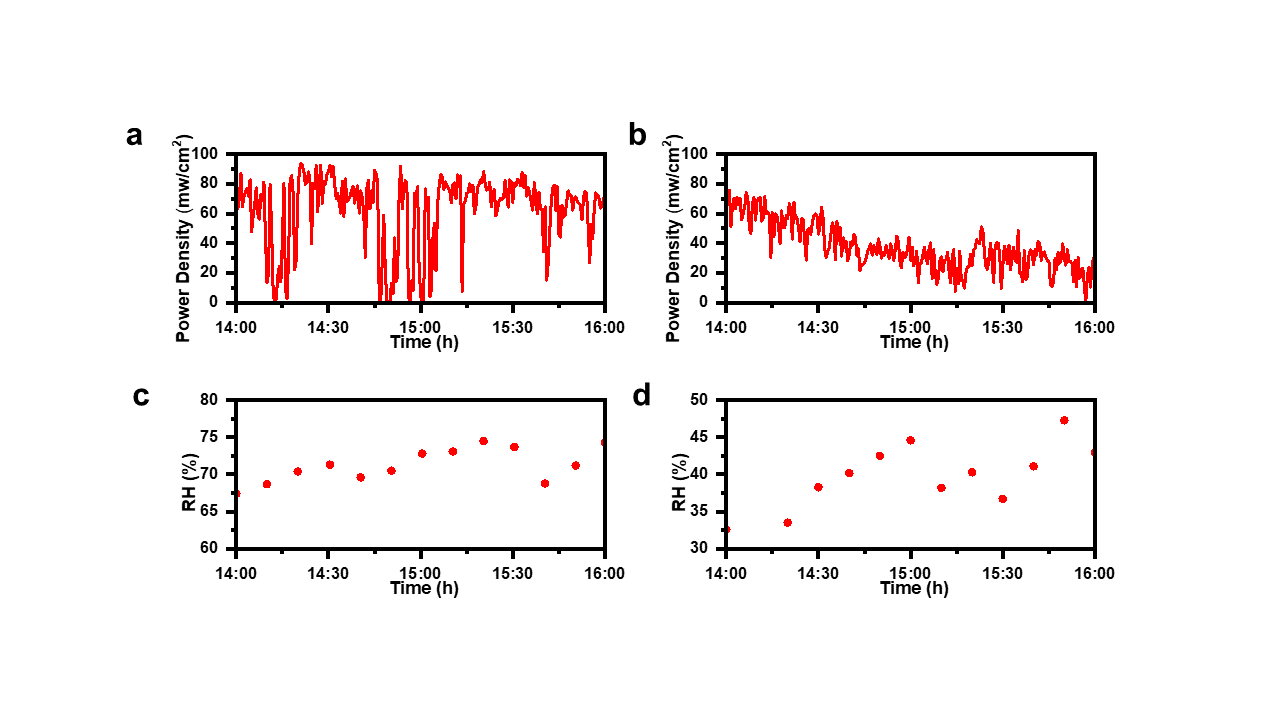


**Figure S15**│**Real-time sunlight power detection** in ShenZhen (a) and Qingdao (b).


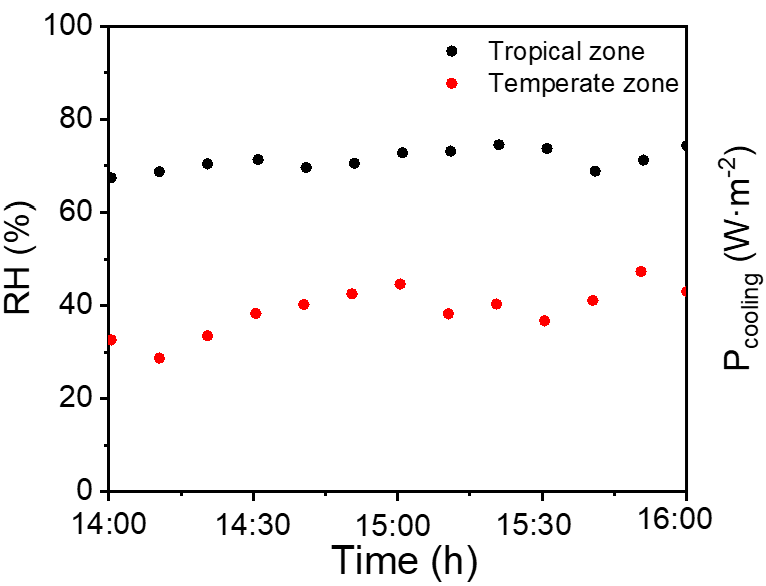


**Figure S16**│**Real-time sunlight power detection** in ShenZhen (a) and Qingdao (b).


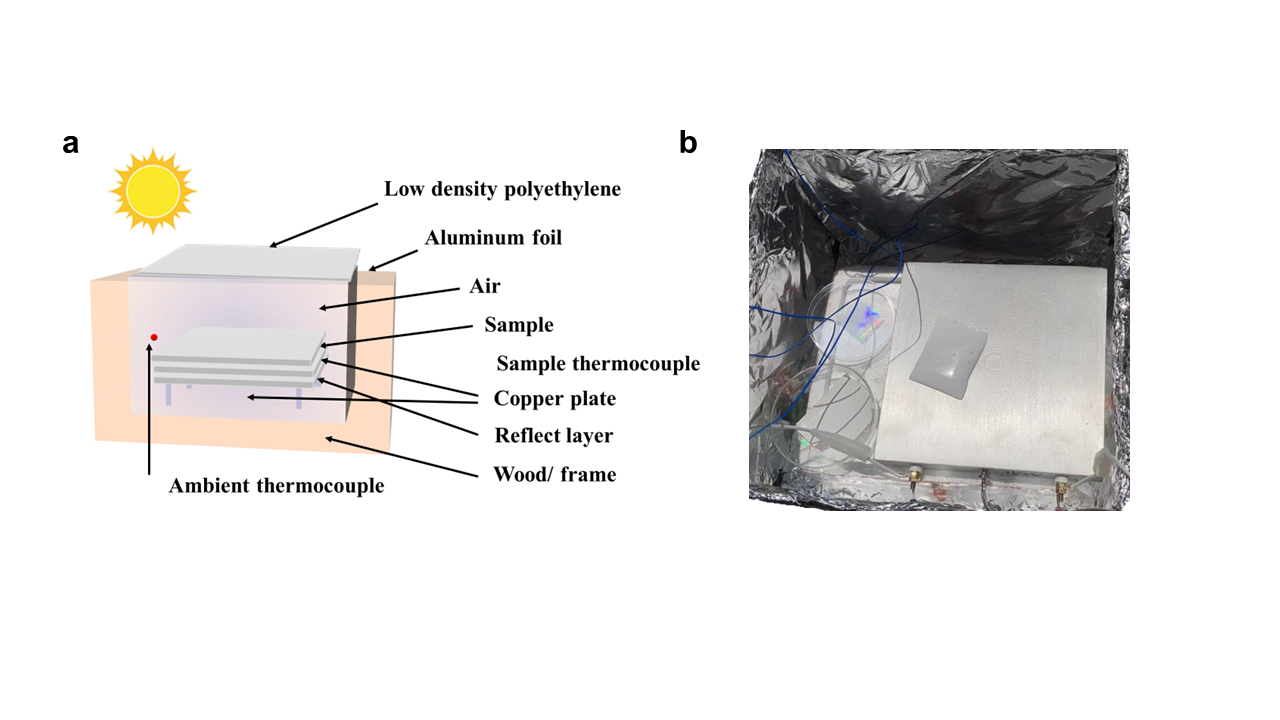


**Figure S17**│**Schematic diagram and optical diagram of thermal compensation experiment for calculating accurate radiative cooling power.** A heatable copper plate was placed under the sample to synchronously control the temperature with the control group.


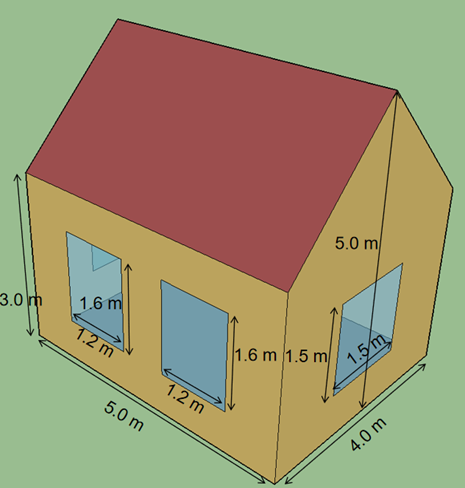


**Figure S18**│ **A Schematic diagram of the model house used in the EnergyPlus simulation.**


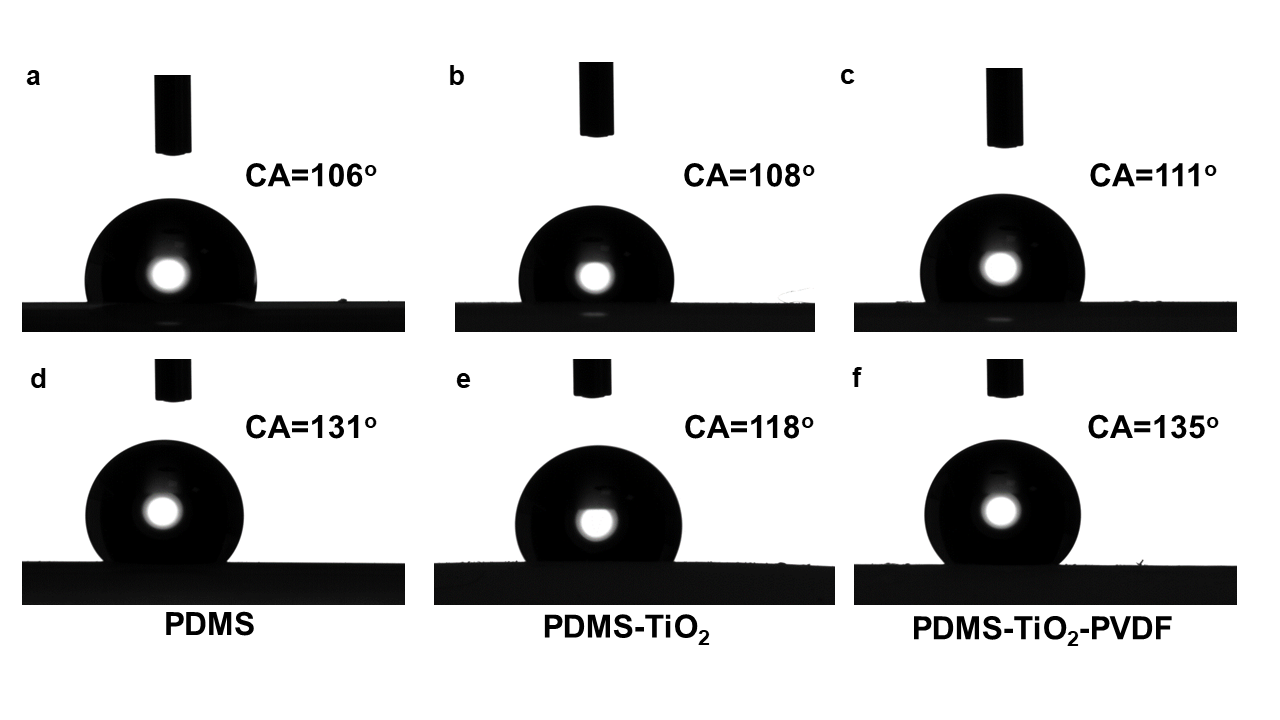


**Figure S19**│**Contact angles between a flat film and the PRCM with the same components. a-c,** flat PDMS films, **d-f,** micro-pyramid array films. From the left to the right are PDMS, TiO_2_ doped PDMS and PRCM.


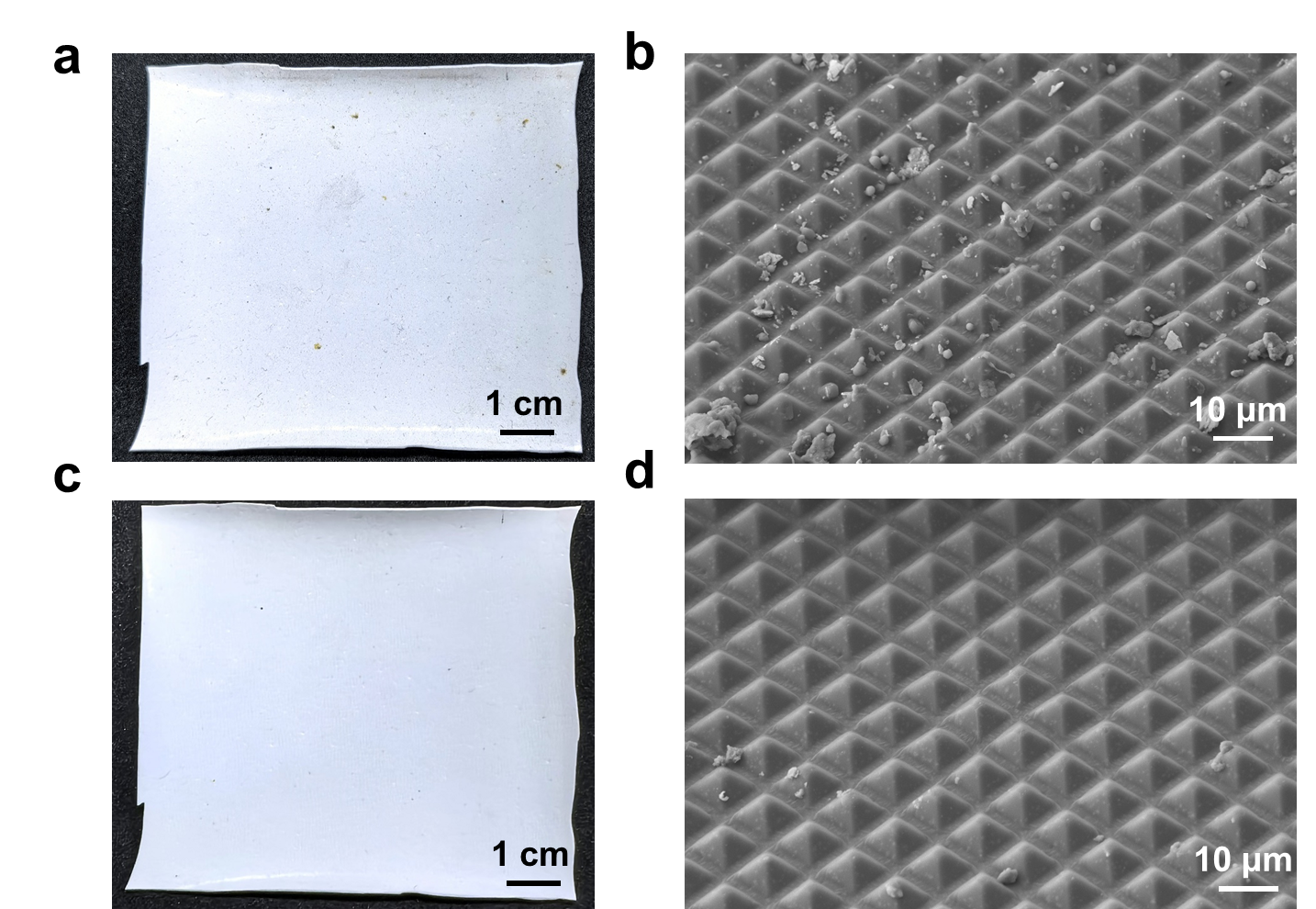


**Figure S20**│**Dust interference experiment.** a, b) Physical and SEM images of the PRCM contained by the dust. c, d) Physical and SEM image of the PRCM washed by the running water.


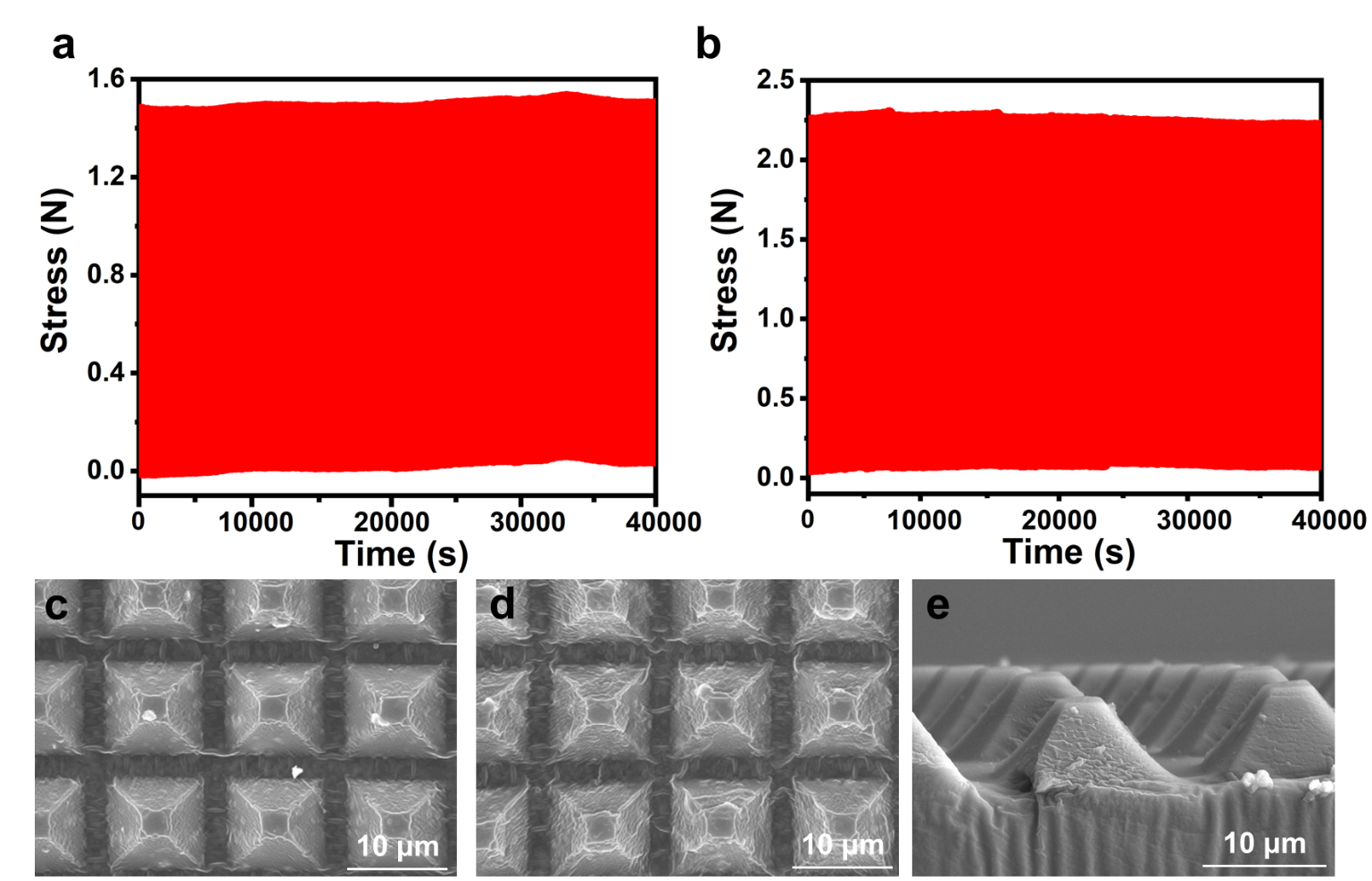


**Figure S21**│**Tensile strength measurement of the membrane.** The stretch elongation is set as 20% of the total length and membrane receive a 10000-cycles-stretching. a) Stress of PRCM in the stretching cycles. b) Stress of pure PDMS with micro-pyramids onside in the stretching cycles. c-e) SEM images of the PRCM. c) before stretching cycles. d) after stretching cycles. e) cross-section after stretching cycles.


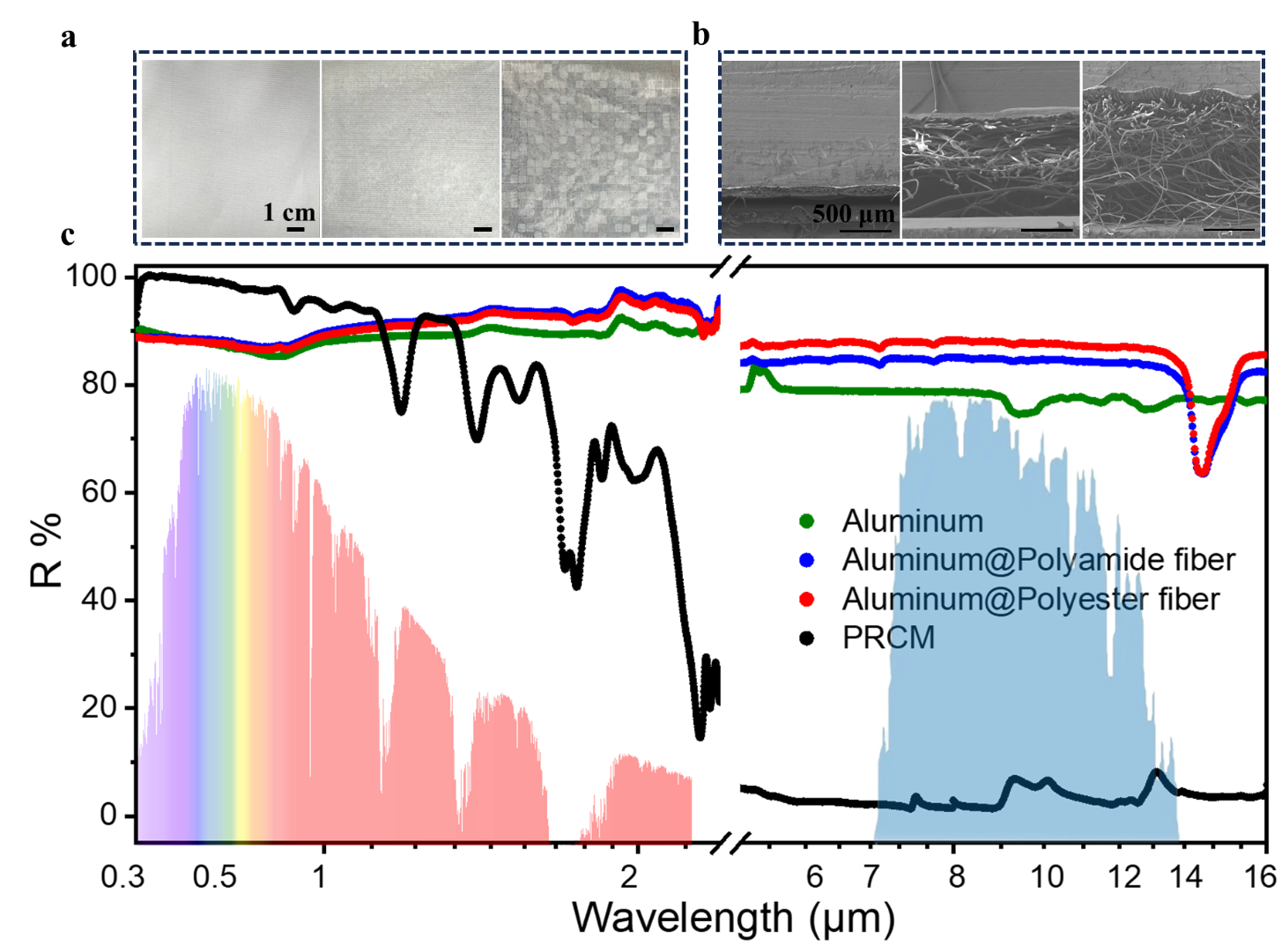


**Figure S22**│**Morphology and optical performance of commercial car clothes.** From left to the right are pure aluminum film, polyester fiber hybrid and polyamide fiber hybrid film respectively. (a) Physical images. (b) Cross-section images. (c) Reflection spectrum detection.


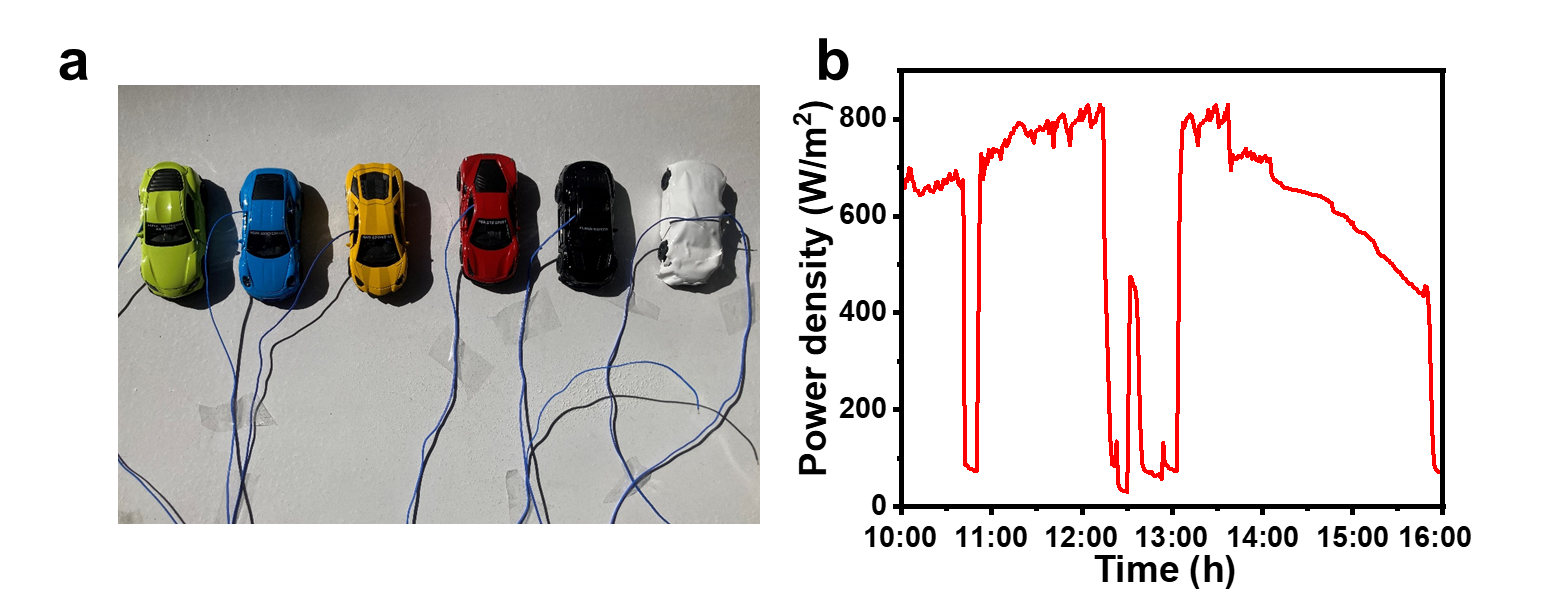


**Figure S23**│**Car model experiment.** a) Photographs of cars in test. Thermocouples are fixed into the interior of the car to detect the inside temperature. b) Real-time temperature records in actual test. The purple line and cyan line record PRCM and room temperature variation respectively while the black, red, yellow, blue and green lines represent the temperature variations inside the car model for the corresponding surficial color respectively. c) Real-time sunlight power density detection.

**Supporting Table**

**Table S1. Optical performance of recent PDRC films.**

| **Number** | **Reference** | **Reflectivity** | **Emissivity** | **Thickness** | |
| --- | --- | --- | --- | --- | --- |
| This work | This work | ~97.3% | ~98.1% | | ~800 μm |
| 1 | *ACS Nano* **2024**, *18*, 11120-11129 | ~96% | > 98% | | > 1000 μm |
| 2 | *Adv. Mater.* **2024**, *36*, 2311633 | 98.3% | 89.2% | | 840 μm |
| 3 | *ACS Nano* **2023**, *17*, 1693-1700 | 97% | 73% | | > 2000 μm |
| 4 | *Solar Energy* **2021**, *225*, 245-251 | > 90% | 94% | | 600 μm |
| 5 | *Adv. Mater.* **2020**, *32*, 2000870 | 95% | 94% | | 600 μm |
| 6 | *Science*, **2019**, *364*, 760 | 92% | 96% | | > 1000 μm |

**Table S2. Optical properties and simulation parameters of PRCM and commercial tile set in the EnergyPlus simulation.**

| **Properties** | **PRCM** | **Commercial Tile** |
| --- | --- | --- |
| Thermal Absorptance | 0.9 | 0.91 |
| Solar Absorptance | 0.028 | 0.24 |
| Visible Absorptance | 0.017 | 0.22 |

**Table S3. Element content of the PRCM.**

| **Elements** | **Weight percentage (%)** | **Atom percentage (%)** |
| --- | --- | --- |
| Silicon (Si) | 44.3 | 28.7 |
| Carbon (C) | 27.6 | 41.7 |
| Oxygen (O) | 23.8 | 26.9 |
| Fluorine (F) | 1.9 | 1.8 |
| Titanium (Ti) | 2.4 | 0.9 |
| Add up | 100 | 100 |

**Table S4. The cost of PRCM ($/m^2^).**

| **Chemicals** | **Price ($/g)** | **Dosage (g/m^2^)** | **Price ($/m^2^)** |
| --- | --- | --- | --- |
| Polydimethylsiloxane (PDMS) | 0.4 | 150 | 60 |
| Titanium dioxide (TiO_2_) | 0.27 | 40 | 10.8 |
| Polyvinylidene fluoride (PVDF) | 1.2 | 10 | 12 |
| Add up |  |  | 82.8 |

**Table S5. Performance comparison between PRCM and commercial car clothes.**

| **Number** | **Materials** | **Thickness (μm)** | **Weight per square (g/m^2^)** | **R_solar_(%)** | **Ɛ_r_ (%)** |
| --- | --- | --- | --- | --- | --- |
| - | PRCM | ~800 | 180-200 | 97.3 | 98.1 |
| 1 | Aluminum | ~100 | 90-110 | 87.8 | 22.7 |
| 2 | Aluminum@Polyamide fiber | ~800 | 130-150 | 88.6 | 17.5 |
| 3 | Aluminum@Polyester fiber | ~1000 | 160-180 | 88.3 | 14.3 |

**References**

1. J. J. He, Q. Y. Zhang, Y. Y. Zhou, Y. Chen, H. X. Ge, S. C. Tang, Bioinspired polymer films with surface ordered pyramid arrays and 3D hierarchical pores for enhanced passive radiative cooling. *ACS Nano* **2024**, *18*, 11120-11129.
2. Y. F. Zhang, J. J. Fu, Y. C. Ding, A. A. Babar, X. Song, F. Chen, X. G. Yu, and Z. J. Zheng Thermal and moisture managing e-textiles enabled by Janus hierarchical gradient honeycombs, *Adv. Mater.* **2024**, *36*, 2311633.
3. K. X. Lin, S. R. Chen, Y. J. Zeng, T. C. Ho, Y. H. Zhu, X. Wang, F. Y. Liu, B. L. Huang, C. Y.-H. Chao, Z. K. Wang, C. Y. Tso, Hierarchically structured passive radiative cooling ceramic with high solar reflectivity. *Science* **2023**, *382*, 691-697.
4. M. Yang, H. M. Zhong, T. Li, B. Y. Wu, Z. K. Wang, D. Z. Sun, Phase change material enhanced radiative cooler for temperature-adaptive thermal regulation. *ACS Nano* **2023**, *17*, 1693-1700.
5. K. Wang, G. L. Luo, X. W. Guo, S. R. Li, Z. J. Liu, C. Yang, Radiative cooling of commercial silicon solar cells using a pyramid-textured PDMS film. *Solar Energy* **2021**, *225*, 245-251.
6. H. X. Zhao, Q. Q. Sun, J. Zhou, X. Deng, J. X. Cui, Switchable cavitation in silicone coatings for energy-saving cooling and heating. *Adv. Mater.* **2020**, *32*, 2000870.
7. T. Li, Y. Zhai, S. M. He, W. T. Gan, Z. Y. Wei, M. Heidarinejad, D. Dalgo, R. Y. Mi, X. P. Zhao, J. W. Song, J. Q. Dai, C. J. Chen, A. Aili, A. Vellore, A. Martini, R. G. Yang, J. Srebric, X. B. Yin, L. B. Hu, A radiative cooling structural material. *Science*, **2019**, *364*, 760.
